# Supplementary material for: Microscopic mechanisms of pressure-induced amorphous-amorphous transitions and crystallisation in silicon
Source: Nat Commun. 2024 Jan 16;15:368. doi: 10.1038/s41467-023-44332-6 (PMC10792069; doi:10.1038/s41467-023-44332-6)
Supplement: Supplementary file 1 — Supplementary Information [file 41467_2023_44332_MOESM1_ESM.pdf]

Supplementary Information for
"Microscopic mechanisms of pressure-induced amorphous-amorphous transitions and crystallisation in silicon"

Zhao Fan[1] and Hajime Tanaka[1,2,*]

[1] *Research Center for Advanced Science and Technology, University of Tokyo, 4-6-1 Komaba, Meguro-ku, Tokyo 153-8505, Japan*

[2] *Department of Fundamental Engineering, Institute of Industrial Science, University of Tokyo, 4-6-1 Komaba, Meguro-ku, Tokyo 153-8505, Japan*

[*] tanaka@iis.u-tokyo.ac.jp

Contents

I. Supplementary Notes 3

II. Supplementary Figures 1-30 12

III. Supplementary Tables 1-2 43

IV. Supplementary References 45

I. Supplementary Notes

Supplementary Note 1: Pressurisation-rate and temperature dependence of structural transitions in a-Si

In our study, we systematically increased the hydrostatic pressure P applied to the as-quenched sample from 0 to 20 GPa using multiple constant rates over the range from 0.01 to 100.0 GPa ps^{-1}, at both 500 and 300 K. The evolution of the average atomic volume and the fraction of different local structural environments with increasing P on the amorphous silicon sample under different conditions is illustrated in Supplementary Fig. 24. Notably, there is almost no structural transition when P is less than 9 GPa under all the conditions considered here (Supplementary Fig. 24m-x).

The occurrence of a structural transition above 9 GPa is dependent on the pressurisation rate. Specifically, at 500 K, there is no structural transition when P is increased up to 20 GPa at a constant rate of 100.0 GPa ps^{-1}. However, at a rate of 10.0 GPa ps^{-1}, a part of LDA environments transforms into HDA environments. At a rate of 1.0 or 0.1 GPa ps^{-1}, the LDA-VHDA transition occurred, similar to what was observed in Ref. [1]. When the pressurisation rate is further reduced to 0.01 GPa ps^{-1}, HDA and VHDA regions sequentially appear before the sample finally crystallises into a mixture of β-Sn and sh structures with increasing P. Therefore, the slower the pressurisation rate, the lower the pressure at which structural transition occurs. When the temperature is reduced from 500 K to 300 K, the main difference is that these structural transitions take place at higher pressures. An interesting observation is that at a rate of 0.1 GPa ps^{-1}, partial crystallisation occurred at 300 K but not at 500 K. This could be attributed to either a high nucleation barrier at 500 K than 300 K or the stochastic nature of the process.

Supplementary Note 2: Influence of sample size on structural transitions in a-Si

We performed an analysis using our local structural order parameters to examine the structural configurations of the ultra-large amorphous silicon model from Ref. [1], which was generated by increasing P from 0 to 20 GPa at 500 K using a constant rate of 0.1 GPa ps^{-1}. The variations in both the volume and fraction of different local structural environments with P are practically identical between the ultra-large sample in Ref. [1] and our sample under the same condition (500 K and 0.1 GPa) (see Supplementary Fig. 24a, m, i and u). The only notable difference is the appearance of crystal nucleation and growth in the ultra-

large sample but not in our sample after the LDA-VHDA transition. This difference is not surprising since a larger sample inherently increases the probability of crystal nucleation. This comparison confirmed that our sample size, consisting of 8,192 atoms, is large enough to study AAT in a-Si. This fact is essential because a relatively smaller sample size enables us to conduct significantly longer timescale simulations, which are crucial for exploring the existence of the monolithic HDA form of this ML-based model a-Si and investigating the mechanism of transitions between the HDA form and the other states.

Supplementary Note 3: Structural transitions occurred at different pressures

In addition to the LDA-HDA transition and subsequent crystallisation occurring at 12 GPa and the LDA-VHDA transition and subsequent crystallisation occurring at 15 GPa (Fig. 1a), we also conducted long timescale isothermal-isobaric relaxation on both the $\mathrm{LDA}_{10,200}$ and $\mathrm{HDA}_{12,1000}$ samples at different pressures after quickly increasing/decreasing the pressure (at a rate of 10 GPa ps^{-1}) on the two samples to different target pressures. From the results shown in Supplementary Figs. 1 and 2, we found the following:

(i) Relaxing the LDA at 12.5 and 13 GPa, the transition sequence is that LDA first transforms into HDA before finally crystallising into a mixture of β-Sn and sh crystals, the same as those observed at 12 GPa. At higher pressure, the LDA-HDA transition and crystallisation occurred earlier; larger VHDA clusters would exist in the resultant HDA; more sh-like atoms would exist in the crystallisation product (see Supplementary Fig. 1a, c).

(ii) Relaxing the LDA at 14 and 15 GPa, the LDA-VHDA transition would occur before crystallising into a sh crystal or a mixture of β-Sn and sh crystals (see Supplementary Fig. 1a, c).

(iii) Relaxing the HDA at 12.5, 13, and 14 GPa, the sample would crystallise into a mixture of β-Sn and sh crystals at 12.5 and 13 GPa or a sh crystal at 14 GPa (see Supplementary Fig. 1b, d).

(iv) Relaxing the HDA at 15 GPa, HDA would first transform into VHDA before finally crystallising into a mixture of β-Sn and sh crystals (see Supplementary Fig. 1b, d).

(v) We released the pressure on the $\mathrm{HDA}_{12,1000}$ sample using two different rates and found, in either case, that the HDA can go back to the initial LDA form, indicating the LDA-HDA transition is reversible, consistent with previous experimental results [2, 3] (see

Supplementary Fig. 25).

(vi) Relaxing the HDA over a pressure range between 7 and 10 GPa, within the timescale we have run (500 ps), we did not observe any AAT, and the slight increase in the sample volume should be due to structural relaxation as the largest LDA clusters always contain less than 300 atoms (see Supplementary Fig. 2c).

(vii) Relaxing the HDA at a pressure $\leqslant$ 6 GPa, the HDA transforms back to LDA partially (see Supplementary Fig. 2a, b).

In brief, we found the following for the three amorphous forms of silicon: LDA is stable when pressure is equal to or less than 6 GPa; the HDA is favourable thermodynamically in the pressure range between 12 and 13 GPa; the VHDA is preferred at further higher pressure. Within the timescale accessible to the current simulations, it is hard to determine if LDA or HDA is more stable over the pressure range between 7 and 11 GPa. It deserves further investigation via powerful supercomputers or advanced simulation techniques. Both HDA and VHDA are easy to crystallise. Significantly, the VHDA is pretty unstable, consistent with the recent observation in ref. [1]. Although the crystallisation product is stochastic to some degree, both HDA and VHDA would crystallise into a mixture of β-Sn and sh structure in most cases, but sh crystal is preferred at higher pressure. We also observed the formation of a single sh crystal at 14 and 15 GPa.

Supplementary Note 4: More details on the structural characteristics of three amorphous forms and their relationship to crystalline structures

We relaxed the as-quenched a-Si sample at 10 GPa for 3 ns and 11 GPa for 2 ns. The average atomic volume decreased by 0.37% at 10 GPa and 0.67% at 11 GPa, respectively (see Supplementary Fig. 3a): a consequence of simple structural relaxation, i.e., pressure-induced elastic shrinkage. We can confirm it in Supplementary Fig. 3b-d; the radial distribution function $g(r)$ uniformly shifts towards the left up to 11 GPa after relaxing for a long time at high pressure compared to the as-quenched a-Si sample at zero pressure. These results corroborate that the LDA form is stable, at least within the timescale accessible to the current MD simulation, when increasing P up to 11 GPa.

When relaxing the as-quenched a-Si at 12 GPa, the sample remains LDA form when pressure just reached to 12 GPa according to its $g(r)$ (see Supplementary Fig. 3d). However, the average atomic volume of the sample decreased by 17.0% (from 17.99 $\mathring{A}^3$ to 14.93 $\mathring{A}^3$)

over the first 500 ps in the subsequent isothermal-isobaric relaxation and dropped again by 2.4% quickly around 1.7 ns (see Fig. 1a). The $g(r)$ of the sample after relaxing 1 ns at 12 GPa is distinctly different from that of the LDA form but without long-range order (see Fig. 1b). Then, we observed long-range order in the sample after relaxing for 2 ns at 12 GPa, and its $g(r)$ is similar to that of bulk β-Sn crystal at the same pressure (Supplementary Fig. 6a). These observations suggest that an AAT from LDA to a different amorphous form and its further crystallisation occurred sequentially during the isothermal-isobaric relaxation at 12 GPa. The CN of the amorphous form obtained at 12 GPa is dominantly CN = 6 (the average CN = 6.09) (see Fig. 1d), and the highest peak on its BADF is located around 90° (see Fig. 1c). These features are consistent with those of HDA form of a-Si revealed in ref. [4], suggesting that the amorphous form obtained at 12 GPa is HDA.

As seen from Fig. 1a, the volume drop magnitude is much larger when increasing pressure from 0 to 15 GPa within 100 ps than when increasing pressure from 0 to 12 GPa. This suggests a structural transformation occurred when increasing pressure from 0 to 15 GPa, besides elastic shrinkage. The volume decreases to its minima ($\sim$ 13.53 $\mathring{A}^3$) around 10 ps and then jumps to a plateau ($\sim$ 13.80 $\mathring{A}^3$). We also confirmed the absence of long-range order in the sample's $g(r)$ after relaxing 10 ps at 15 GPa, and the $g(r)$ is different from either the initial LDA form or the new form that appeared at 12 GPa. As seen from Fig. 1b, compared to the amorphous form that appeared at 12 GPa, the position of the first peak of the new amorphous form at 15 GPa moved further towards the right side; the intensity of the second peak decreased greatly while the third peak surges around the shoulder position of the third peak of $g(r)$ of the amorphous form at 12 GPa (HDA) and the intensity of the third peak is even higher than that of its second peak. Moreover, the $g(r)$ of the sample at the end of the relaxation is closer to that of bulk sh crystal at the same pressure (see Supplementary Fig. 6a).

These results suggest that the initial LDA form transforms into another amorphous form distinct from HDA when pressure is increased from 0 to 15 GPa. The structural transition finished after relaxing the sample at 15 GPa for a short time ($\sim$ 10 ps). This amorphous form that appeared at 15 GPa is unstable and quickly crystallises during the subsequent relaxation. The CN of the amorphous form obtained at 15 GPa is dominantly CN = 8 (the average CN = 8.21) (see Fig. 1d), and the highest peak on its BADF is located around 60° (see Fig. 1c). These features are consistent with those of the VHDA form of a-Si revealed in

refs. [4, 5]. Note that Durandurdu and Drabold termed the amorphous form they observed at 16.25 GPa HDA [5], and later Morishita pointed out that this amorphous form should be VHDA [4]. Thus, the amorphous form we observed at 15 GPa should be VHDA.

The density and CN distribution are the most pronounced differences among the three amorphous forms. The noticeable difference in the CN distribution among the three amorphous forms should be due to different local atomic environments in different amorphous forms rather than pressure-induced elastic shrinkage, although we used a constant cutoff of 2.85 Å, the same as in ref. [1], to determine CN. The variation of the CN distribution due to elastic shrinkage under pressure is trivial (see Supplementary Fig. 26). Other structural analyses further support this conclusion. We can use a single coarse-grained local bond orientational order parameter $\bar{q}_4^{21}$ (see Methods for the definition) to identify local LDA-like environments from the other two local amorphous environments (see Supplementary Fig. 4d). Furthermore, a convolutional neural network (CNN) model [6, 7] can separate HDA- and VHDA-like environments with an accuracy of $> 99.66\%$ (Supplementary Fig. 4f, see Methods for the details).

Through ring analysis [8], we found that the local atomic environments in the LDA and VHDA forms are dominated by 6- and 4-membered rings, respectively, regardless of their CN. On the other hand, for the HDA form, the networks centred on atoms with CN ≤ 6 mainly consist of 5-membered rings, and those with CN > 6 are dominated by 4-membered rings, as shown in Supplementary Fig. 10.

Besides the difference in structure among the three amorphous forms discussed above and in the main text, there are other interesting differences in their density relative to that of their crystalline counterparts and the shift of the first peak of $g(r)$ with increasing pressure. Specifically, at 0 GPa, either cubic- or hexagonal-diamond crystal is denser than the LDA form of silicon, and at 12 GPa, either β-Sn or sh crystal is denser than the HDA form. In contrast, the VHDA form at 15 GPa is denser than either β-Sn or sh crystal at the same pressure. We note that the LDA form can be denser than either cubic- or hexagonal-diamond crystal at 10 GPa, perhaps because more HDA-like atoms exist in the LDA form at 10 GPa.

We observed that the first peak of $g(r)$ of LDA samples shifts towards the left side with increasing pressure while the first peak of both HDA and VHDA samples shift towards the right side, as shown in Supplementary Fig. 11a-c, although the volume decreases with increasing pressure for all the three amorphous forms. As seen from Supplementary Fig. 11d-

l, atoms with larger CN have smaller atomic volumes but longer bond lengths in all three amorphous forms. Moreover, increasing pressure results in a slight variation in the CN distribution of LDA samples but a considerable variation for the two dense forms. In addition, with increasing pressure on LDA samples, there is a visible shift towards the left for the first peak of $g(r)$ and a volume decrease for atoms with identical CN. In contrast, applying pressure can result in visible variation in atomic volume but has little influence on the first peak of $g(r)$ for atoms with identical CN in the two dense forms. These should be responsible for the opposite shift behaviour of the first peak of $g(r)$ with pressure between the LDA form and the other two dense forms. In other words, increasing hydrostatic pressure leads mainly to elastic shrinkage on LDA samples but structural rearrangements (variation of CN distributions) on the two dense forms, indicating the higher stability of local tetrahedral structures of LDA with smaller distortions.

Supplementary Note 5: Further characterisation of the HDA local structure

It is well known that the locally favoured structure in the LDA form of silicon is a regular four-folded polyhedron—tetrahedron [9, 10], which is coincidently the local polyhedron in its crystalline counterpart—diamond structure. Given that the dominant CN in the HDA form is 6, and the highest peak of the BADF of the HDA form is located around 90° (see Fig. 1c, d). Thus, it is natural to hypothesise that the locally favoured structure in the HDA form is a six-folded regular polyhedron—octahedron. However, the perfect local polyhedron in a bulk β-Sn crystal, which is the crystalline counterpart of the HDA form of silicon [4], is also very close to a standard octahedron. Supplementary Fig. 27 illustrates a perfect local polyhedron in a bulk β-Sn crystal, and Supplementary Table 2 lists the corresponding spherical coordinates. To determine whether the locally favoured structure in the HDA form is a perfect local β-Sn polyhedron or an octahedron, we calculated the minimised deviation of local environments centred on atoms with CN = 6 in the $\text{HDA}_{12,1000}$ sample from both a perfect local β-Sn polyhedron and an octahedron through both permutation and rotation. Specifically, we used the minimised root mean squared displacement (RMSD) to measure the degree of deviation of an actual local polyhedron from the template (a perfect polyhedron). The minimised RMSD between an actual local polyhedron α centred on atom

i and a templated polyhedron β is defined as

$$\Delta_{i,\beta} = \frac{1}{N}\sqrt{\min_{\mathbb{R}_i,\mathbb{P}_i}(\sum_{j=1}^{N}|\hat{r}_{ij,\beta} - \mathbb{R}_i\mathbb{P}_i\hat{r}_{ij'}|^2),}, \quad (1)$$

where $\hat{r}_{ij,\beta}$ is a unit vector connecting the central atom i to atom j, one of the N neighbours in a templated polyhedron β (the templated polyhedron β can be any specified polyhedron of interest, such as a standard octahedron or perfect local β-Sn polyhedron). $\hat{r}_{ij'}$ is the unit vector connecting the central atom i of an actual local polyhedron α to one of its first N nearest neighbours, j'. Note that j' is unnecessary to be equal to j but depends on the permutation operation $\mathbb{P}_i$. $\mathbb{R}_i$ is a rotation operation. Note that unit vectors are used since we solely consider the bond-orientational difference here. As seen from Supplementary Fig. 9b, which shows the distributions of RMSD of local atomic environments centred on atoms with CN = 6 in the HDA form relative to both a perfect local β-Sn polyhedron and an octahedron, the degree of deviation of local symmetry of HDA relative to a regular octahedron and local β-Sn is very similar, but smaller relative to a local β-Sn. However, this result may be due to inevitable octahedral symmetry distortion. Thus, we infer that the locally favoured structure of HDA has octahedral orientational symmetry.

Supplementary Note 6: Comparing local atomic environments centred on atoms with the same CN in HDA and VHDA

As shown in Supplementary Fig. 9b, c, the deviation from either a perfect local β-Sn polyhedron or a regular octahedron is comparable between six-folded polyhedra in the VHDA form, and those in the HDA form and the deviation from a perfect local sh polyhedron is comparable between eight-folded polyhedra in the HDA form and those in the VHDA form. These suggest that the local atomic environments centred on atoms with the same CN are comparable between the HDA and VHDA forms (although there is some difference from the standpoint of ring analysis, see Supplementary Fig. 10). We also compared both $g(r)$ and BADF of atoms with the same CN between the HDA and VHDA forms (see Supplementary Fig. 8), providing further support that the local atomic environments centred on atoms with the same CN are approximately comparable between the HDA and VHDA forms. Thus, the main difference between the HDA and VHDA forms is the fraction of atoms with different CNs.

Supplementary Note 7: LDA-HDA transitions observed at 12.5 and 13 GP

The LDA-HDA transitions at 12.5 and 13 GPa also occurred through NG (see Supplementary Fig. 17). We used two protocols to increase pressure before the isothermal-isobaric relaxation at 12.5 and 13 GPa. In case 0 (denoted in Supplementary Fig. 1a), the pressure was first increased linearly from 0 to 10 GPa within 100 ps, and then the pressure was kept constant at 10 GPa for 200 ps before it is finally increased to 12.5 or 13 GPa at a rate of 10 GPa ps^{-1}, i.e., the $LDA_{10,200}$ sample was used in the case 0. In case 1 (also denoted in Supplementary Fig. 1a), the pressure was increased directly from 0 to 12.5 or 13 GPa at a rate of 10 GPa ps^{-1} on the as-quenched sample. In both cases, the LDA-HDA transitions occurred through NG. This suggests that the NG mechanism we observed in the LDA-HDA transition does not depend on the protocol of increasing pressure.

Supplementary Note 8: Other HDA-LDA transitions

We also examined the HDA-LDA transitions, which take place during continuously reducing pressures and isothermal-isobaric relaxation at higher pressures. We found that all these HDA-LDA transitions proceed via SD (see Supplementary Figs. 2 and 25). Only roughly 80%, 60%, and 30% atoms have local tetrahedral order for isothermal-isobaric relaxation at 4, 5, and 6 GPa, respectively (see Supplementary Fig. 2b). Despite only 30% of the atoms exhibiting tetrahedral order at 6 GPa, they still form a network-like supercluster that spans the supercell, rather than organising into several discrete LDA-like domains. This behaviour should be the consequence of SD.

Supplementary Note 9: LDA-VHDA transitions induced by increasing pressure continuously

We conducted structural analysis on the snapshots during the LDA-VHDA transitions induced by increasing pressure continuously from 0 to 20 GPa at different pressurisation rates and/or temperatures. As seen from Supplementary Figs. 24g-l and 28, VHDA nuclei emerge exclusively inside the HDA regions, the same as that observed during the LDA-VHDA transitions under isothermal-isobaric conditions (see discussion in the main text).

We also analysed the structural snapshots from ref. [1] about the amorphous-amorphous transition observed on an ultra-big sample (100,000 atoms) when increasing pressure from 0 to 20 with a rate of 0.1 GPa ps^{-1} at 500 K using the local structural order parameters developed in the current work. As presented in Supplementary Fig. 29a, there is almost

no structural transition when P is not greater than 10 GPa, and several large HDA-like clusters formed in the LDA sample when P was increased upto 11 GPa. Then, the VHDA-like clusters initially appeared within the HDA-like region at 12 GPa. We confirmed that all atoms within the first neighbouring shell surrounding these VHDA clusters are HDA-like atoms at 12 GPa. These VHDA-like clusters then quickly grew and dominated the entire supercell. Accompanying the appearance of VHDA form, several crystal nuclei emerged. These processes are almost identical to those we observed in our sample under the same conditions.

Therefore, the LDA-VHDA transitions induced by increasing pressure continuously also proceed through an intermediate HDA state. In other words, initial VHDA clusters do not appear directly from LDA regions but from the intermediate HDA regions.

Supplementary Note 10: Crystallisation behaviours of a-Si induced by increasing pressure continuously

As seen from Supplementary Fig. 30, during the crystallisation processes induced by continuously increasing pressure, β-Sn-like clusters always appeared earlier than sh-like clusters and sh-crystal nuclei initiated within the core of β-Sn-like clusters. In addition, Supplementary Fig. 29b visualises the crystallisation process of the ultra-big a-Si sample in ref. [1] using our local order parameters. Notably, for this ultra-large sample, β-Sn-like atoms always appeared in the interface between crystal nuclei and amorphous regions during crystal nucleation and growth and also occupied the peripheral area of final crystalline grains. These results also suggest that β-Sn crystal is an intermediate state in the crystallisation of a-Si into sh crystal under pressure.

II. Supplementary Figures 1-30

Source data for all the figures, excluding image data, is available in the Source Data Files.

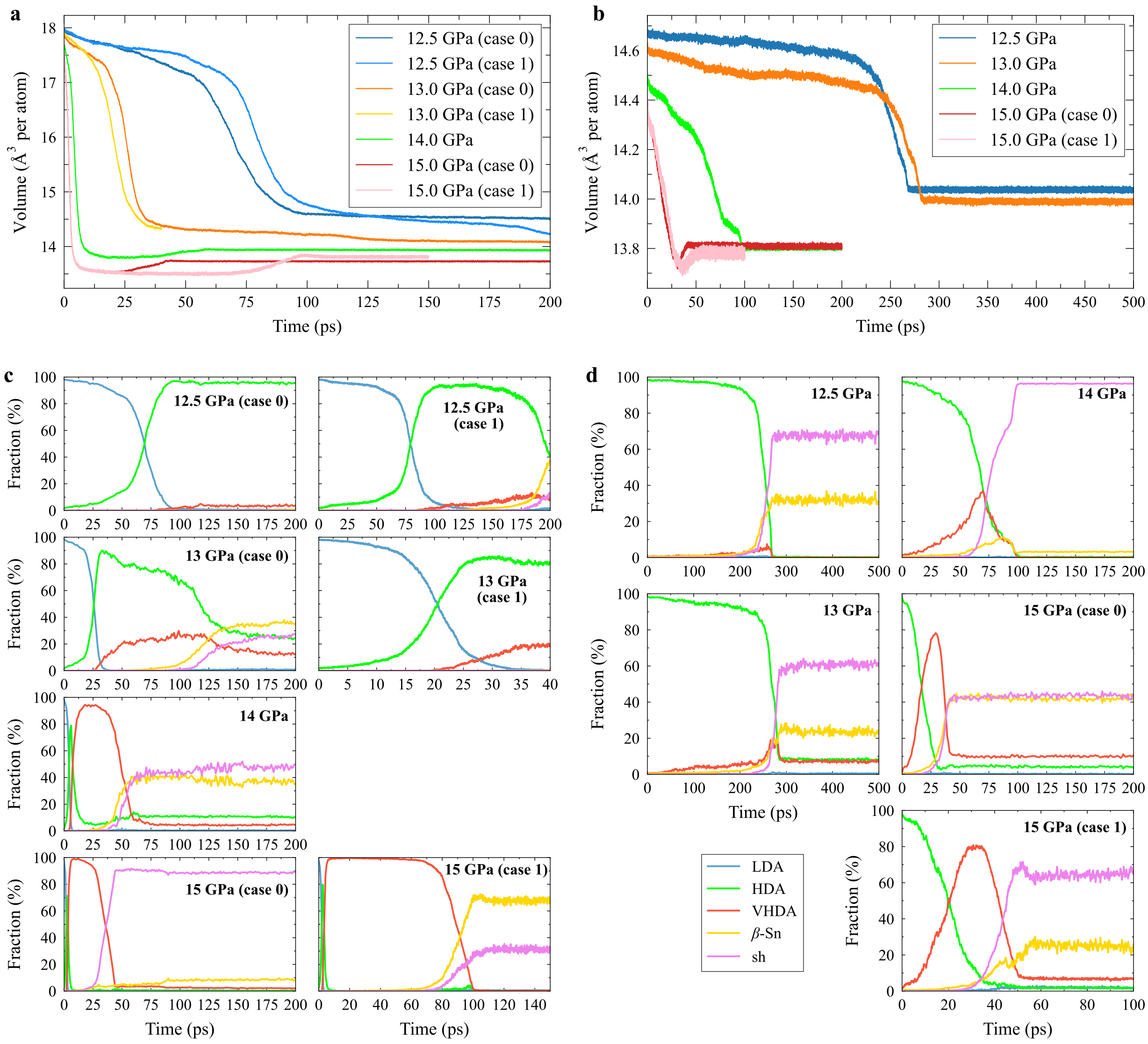

Supplementary Fig. 1. Isothermal-isobaric relaxation on both low-density amorphous (LDA) and high-density amorphous (HDA). a and **b** show the time evolution of the average atomic volume when annealing the LDA$_{10,200}$ and HDA$_{12,1000}$ samples, respectively, at 300 K for different pressures. **c** and **d** show the temporal change of the fraction of the five different local structural environments (LDA, HDA, very-high-density amorphous (VHDA), β-Sn, and simple hexagonal (sh)) when annealing the LDA and HDA samples, respectively. In each case, pressure on the initial sample was quickly increased to different target pressures at a constant rate of 10 GPa ps^{-1} before the isothermal-isobaric relaxation. At 15 GPa, the only difference between case 0 and case 1 is the different number of CPU cores used in the simulations. Two initial LDA samples were used when conducting isothermal-isobaric relaxation at 12.5 and 13 GPa. In case 0, the initial sample is the LDA$_{10,200}$ sample; in case 1, the initial sample is the as-quenched sample, and the pressure on it was increased quickly from 0 to the specified pressure at a constant rate of 10 GPa ps^{-1} before the isothermal-isobaric relaxation.

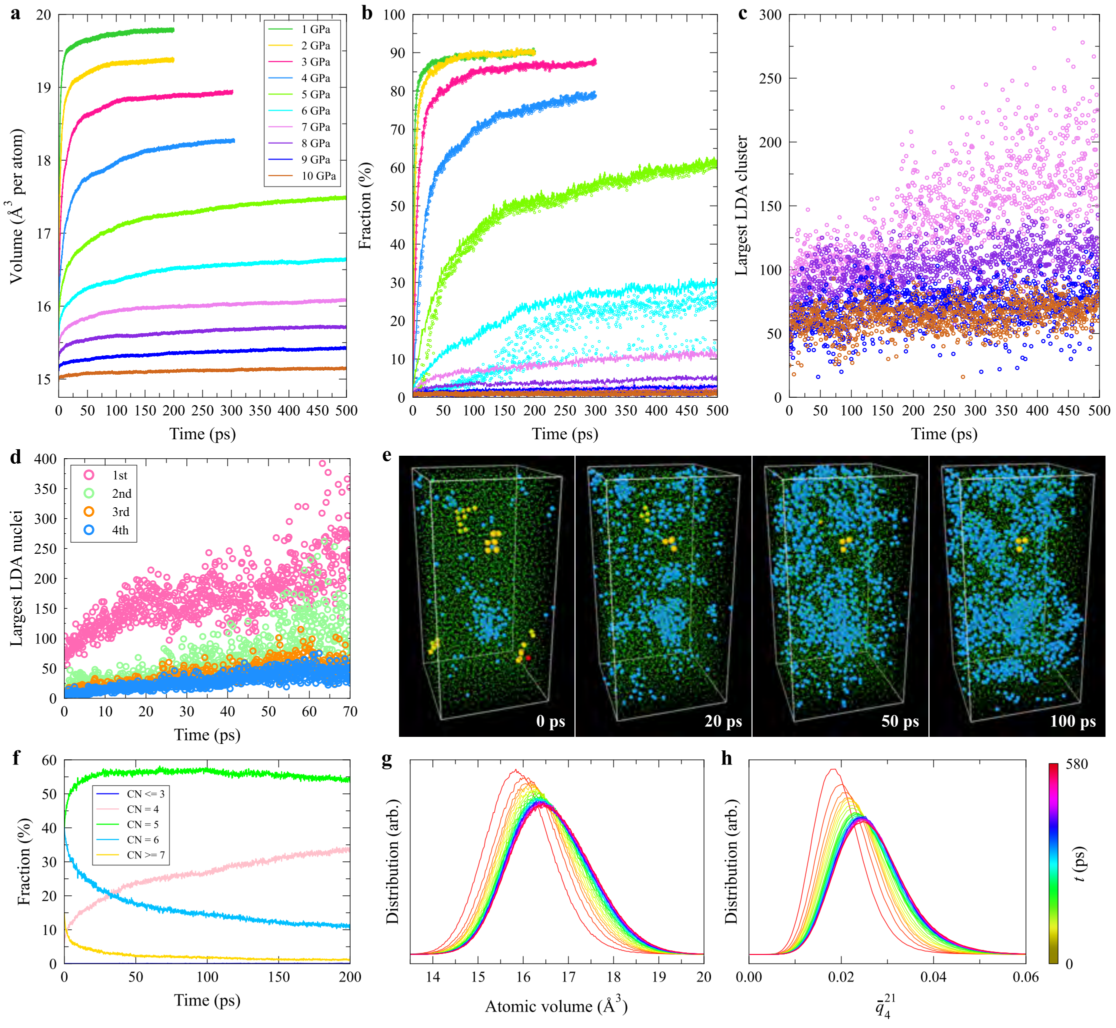

Supplementary Fig. 2. Relaxing high-density amorphous (HDA) at low pressures. a and **b** show the evolution of the average atomic volume and the fraction of low-density amorphous (LDA)-like atoms, respectively, when conducting isothermal-isobaric relaxation on the $HDA_{12,1000}$ sample at different pressures $\leqslant$ 10 GPa. The pressure on the $HDA_{12,1000}$ sample was first decreased from 12 GPa to different target values at a constant rate of 10 GPa ps^{-1} before the isothermal-isobaric relaxation. The open circles in **b** represent the number ratio of the atoms in the largest LDA cluster over the atoms in the supercell box. **c** highlights the variation of the largest LDA cluster size when relaxing the HDA sample at relatively high pressures (7-10 GPa). **d-h** depict the HDA-LDA transition behaviour at 6 GPa, which occurs when relaxing the $HDA_{12,1000}$ sample at 6 GPa. **d** The size evolution of the first several largest LDA nuclei. A 2.85 Å cutoff was used for cluster analysis. **e** Typical structural snapshots during the HDA-LDA transition. Blue, green, red, and yellow spheres represent LDA-, HDA-, very-high-density amorphous (VHDA)-, and β-Sn-like atoms, respectively. There is no simple hexagonal (sh)-like atom during the process considered here. The atom size is adjusted for different structural types for clarity. **f**, **g**, and **h** show the variation of the coordination number (CN) distribution, atomic volume distribution, and distribution of coarse-grained local bond orientational order parameter $\bar{q}_4^{21}$, respectively, during the transition.

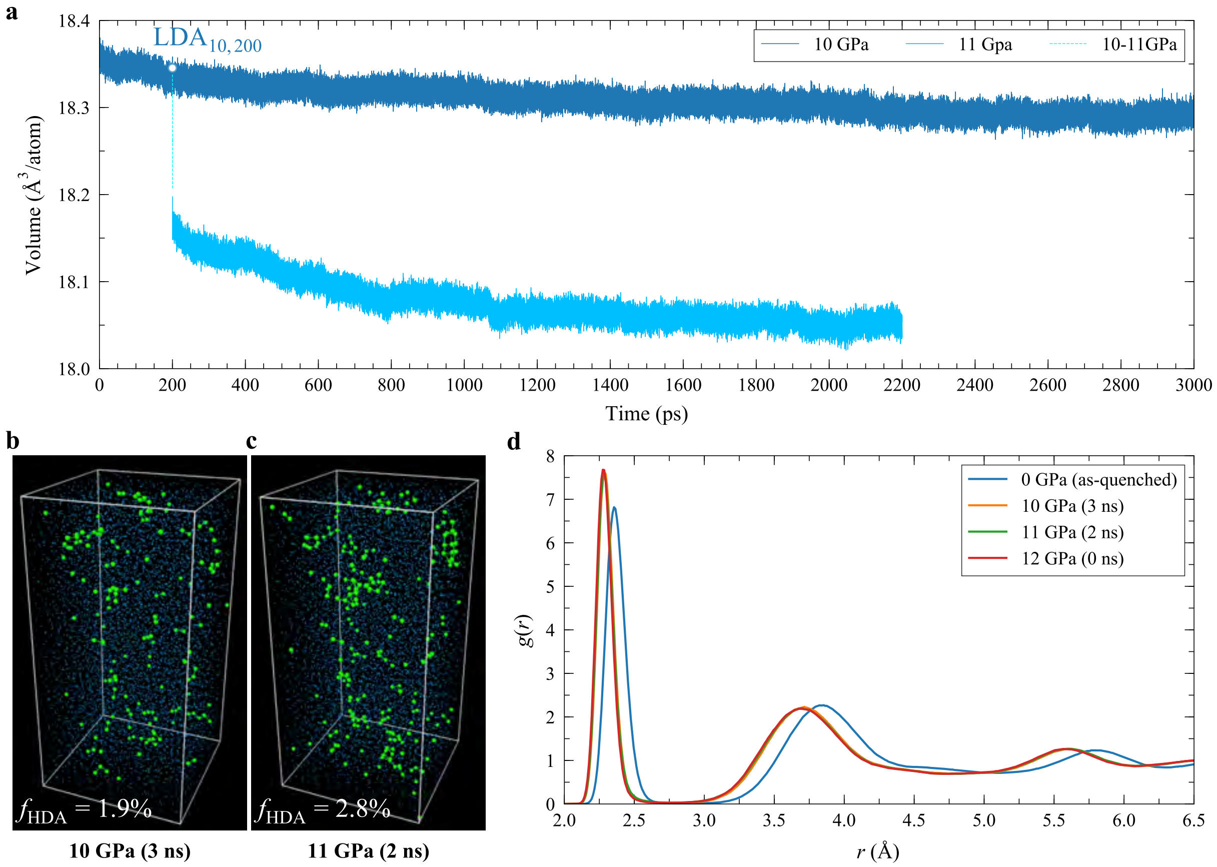

Supplementary Fig. 3. Stability of low-density amorphous (LDA) below 11 GPa. a The temporal variation of the average atomic volume at 300 K when relaxing the as-quenched a-Si at 10 GPa for 3 ns. The sample is also relaxed for 2 ns at 11 GPa after quickly increasing pressure (10 GPa ps^{-1}) from 10 to 11 GPa, as shown in **a**. **b** and **c** show the final structural snapshot of a-Si after relaxing for 3 ns at 10 GPa and for 2 ns at 11 GPa, respectively. Blue and green spheres represent LDA- and high-density amorphous (HDA)-like atoms, respectively. There are no other types of atoms. The fraction of HDA-like atoms (f_{HDA}) is denoted on the corresponding panel. The atom size is adjusted for different structural types for clarity. **d** Comparing the radial distribution function $g(r)$ of a-Si samples after relaxing at different pressures to that of the initial as-quenched a-Si at 0 GPa. The temperature is always at 300 K.

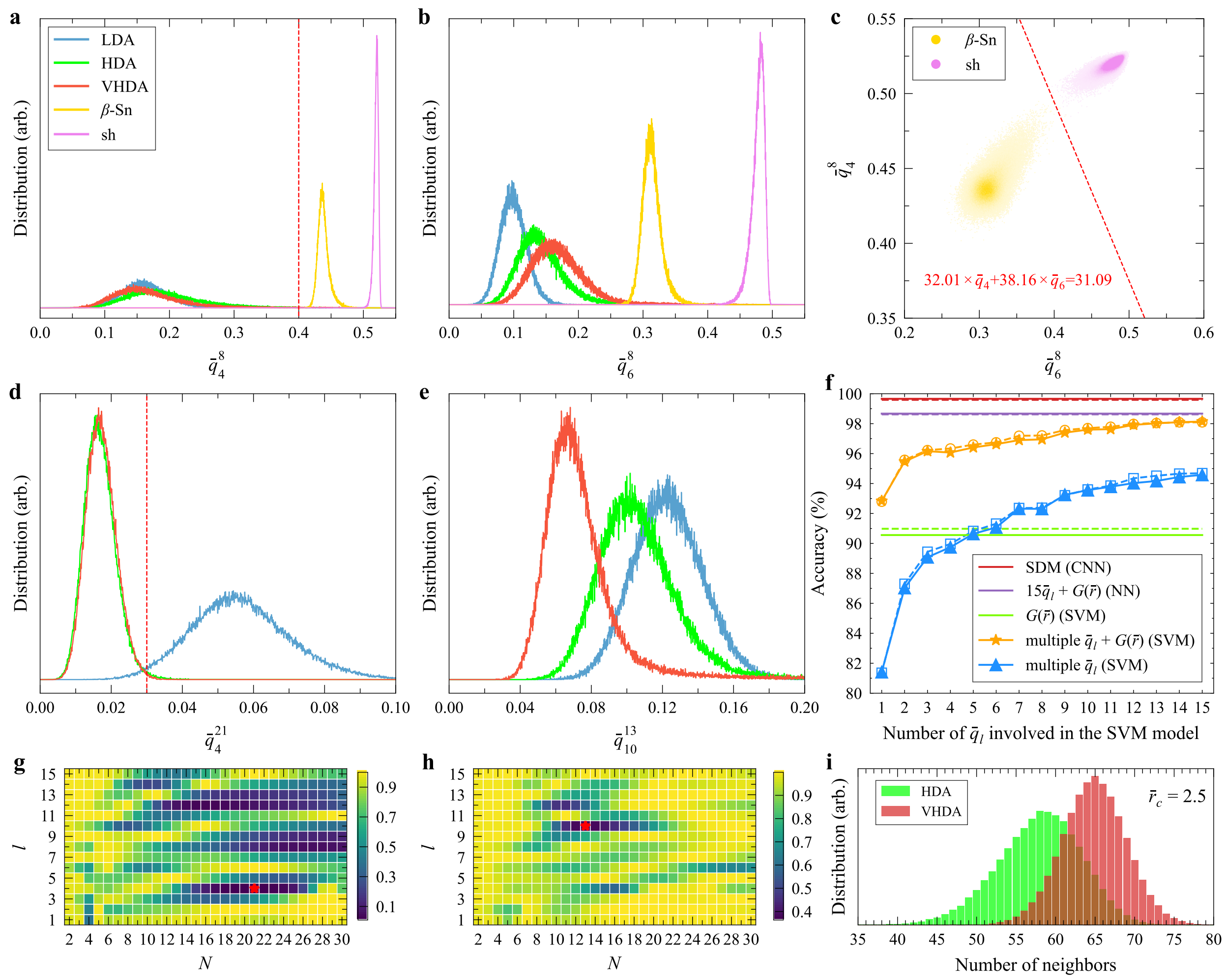

Supplementary Fig. 4. Identifying different local structural environments. **a** and **b** show the distributions of coarse-grained local bond orientational order parameter $\bar{q}_4^8$ and $\bar{q}_6^8$, respectively, for the three amorphous forms, i.e., low-density amorphous (LDA), high-density amorphous (HDA), and very-high-density amorphous (VHDA), as well as bulk β-Sn and bulk simple hexagonal (sh) crystals. The red vertical dashed line in **a** denotes the threshold of $\bar{q}_4^8$, 0.4, which is used to differentiate crystal-like environments from the three amorphous environments. **c** shows the density of local atomic environments from both bulk β-Sn and sh crystals in the $\bar{q}_4^8$-$\bar{q}_6^8$ plane. Darker colour corresponds to higher density. The red dashed line in **c** represents the best boundary separating β-Sn- and sh-like environments. **d** and **e** show the distributions of $\bar{q}_4^{21}$ and $\bar{q}_{10}^{13}$, respectively, for the three amorphous forms. The red vertical dashed line in **d** denotes the threshold of $\bar{q}_4^{21}$, 0.03, which is used to differentiate LDA-like environments from both HDA- and VHDA-like environments. **f** The accuracy of various machine learning models in separating local structural environments in VHDA from those in HDA. The solid lines (solid symbols) and dashed lines (empty symbols) represent test and training accuracy, respectively. **g** and **h** show the dependence of $O_{\mathrm{LDA-HDA}}$ and $O_{\mathrm{HDA-VHDA}}$, respectively, on both the two free parameters l and N. $O_{\alpha-\beta}$ quantifies the degree of overlap in the distributions of $\bar{q}_l^N$ between two structural forms, α and β. See Methods for the definition of $O_{\alpha-\beta}$. The red star in each panel marks the lowest $O_{\alpha-\beta}$. **i** The distributions of the number of neighbours within the reduced cutoff of 2.5 for both HDA and VHDA samples.

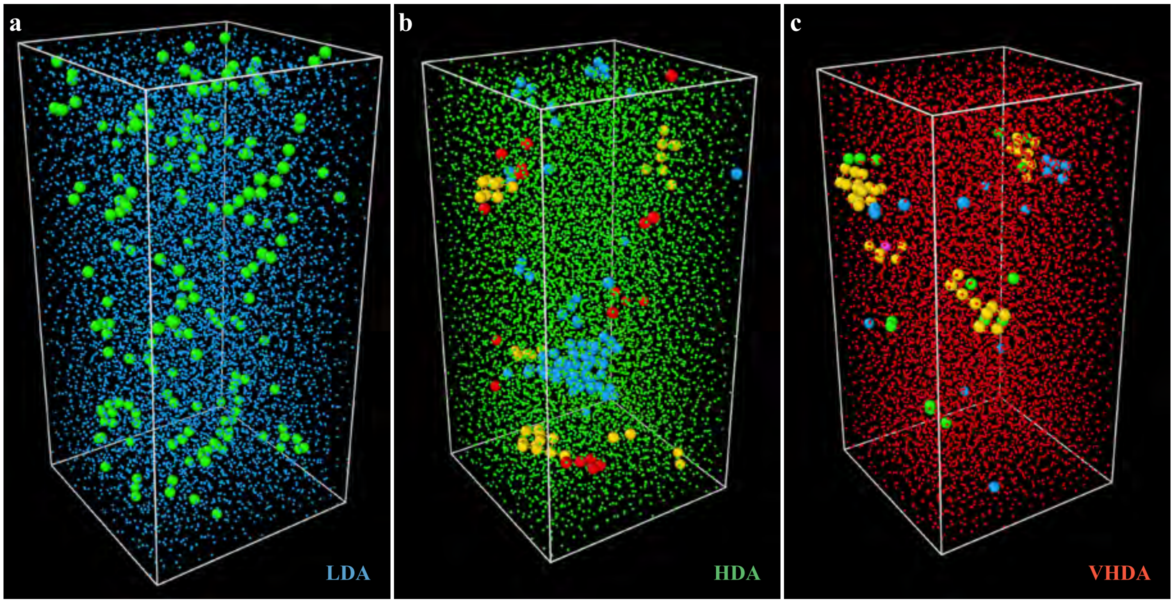

Supplementary Fig. 5. Typical structural configurations of the three amorphous forms of silicon. **a**, **b**, and **c** represent the structural snapshot of the low-density amorphous ($LDA_{10,200}$), high-density amorphous ($HDA_{12,1000}$), and very-high-density amorphous ($VHDA_{15,10}$) samples, respectively, at 300 K. Blue, green, red, yellow, and magenta spheres represent LDA-, HDA-, VHDA-, β-Sn-, and simple hexagonal (sh)-like atoms, respectively. The atom size is adjusted for different structural types for clarity.

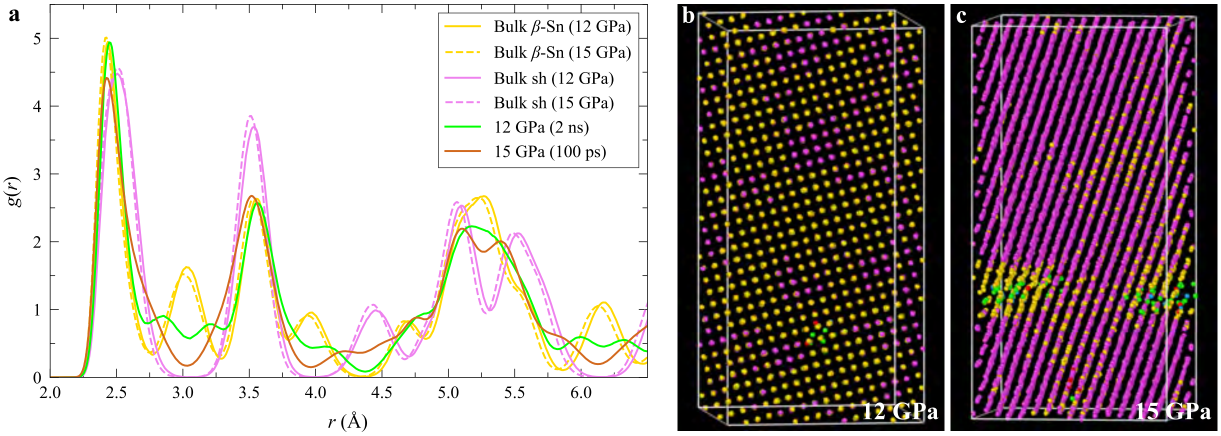

Supplementary Fig. 6. Crystallisation products of the as-quenched a-Si after relaxation at both 12 and 15 GPa. **a** The radial distribution function $g(r)$ of the sample after relaxing the as-quenched a-Si at 12 GPa for 2 ns and at 15 GPa for 100 ps, respectively, as well as bulk β-Sn and simple hexagonal (sh) crystals at both 12 and 15 GPa. **b** and **c** show the final structural snapshot after relaxing at 12 and 15 GPa, respectively. Blue, green, red, yellow, and magenta spheres represent low-density amorphous (LDA)-, high-density amorphous (HDA)-, very-high-density amorphous (VHDA)-, β-Sn-, and sh-like atoms, respectively.

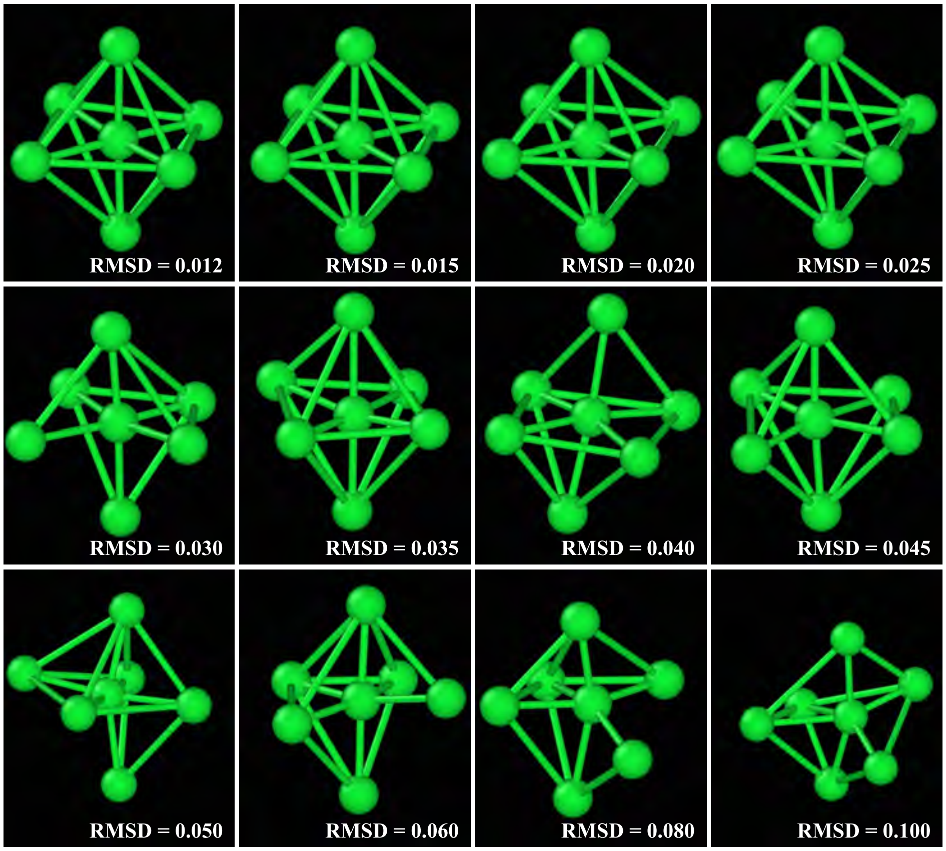

Supplementary Fig. 7. Characteristic octahedral environments in high-density amorphous (HDA). Typical octahedral environments with different RMSD relative to a perfect octahedron surrounding atoms with a coordination number (CN) of 6 in the HDA form of a-Si. Here, the pairs of atoms with an interatomic distance $\leqslant 3.72$ Å are connected with a bond. See Supplementary Note 5 for the definition of RMSD.

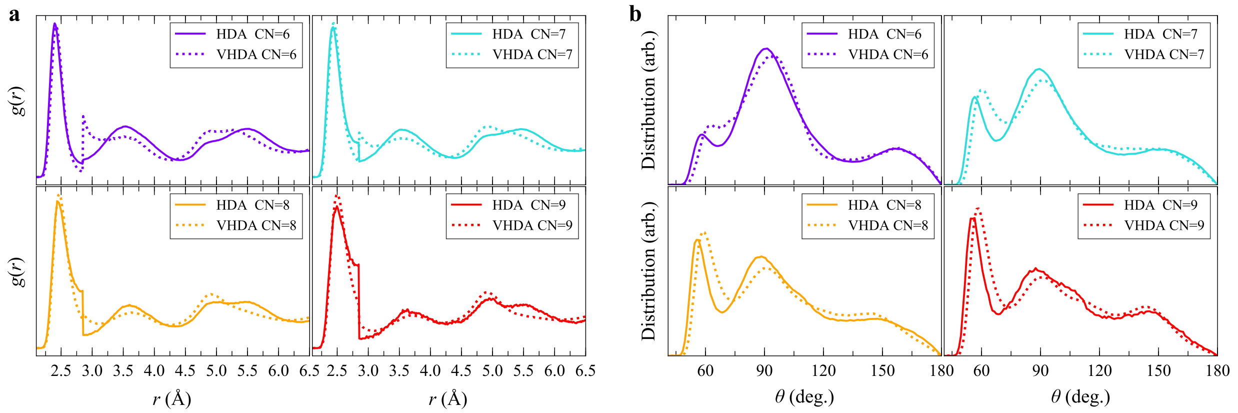

Supplementary Fig. 8. Structural differences between high-density amorphous (HDA) and very-high-density amorphous (VHDA). Comparison of the radial distribution function $g(r)$ (**a**) and bond angle distribution function (BADF) (**b**) for atoms with the same coordination number (CN) between the HDA and VHDA forms.

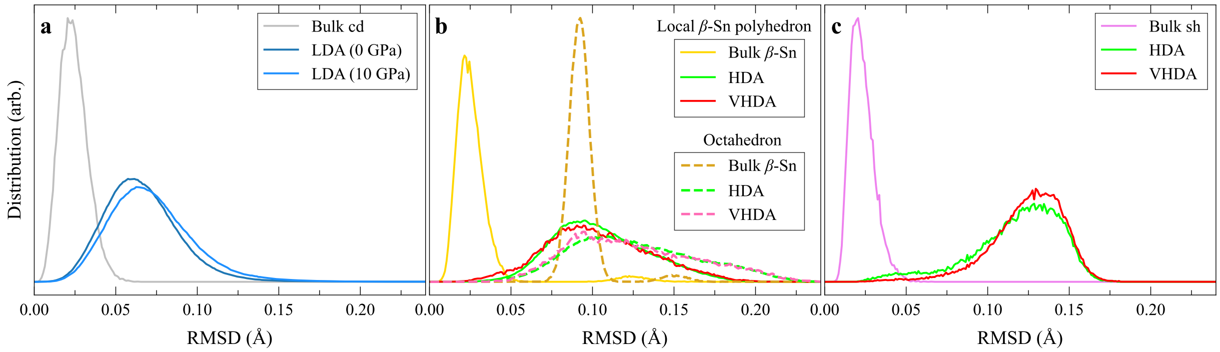

Supplementary Fig. 9. Similarity of local polyhedra in the three amorphous forms to several given polyhedra. **a** The distribution of the root mean squared displacement (RMSD) (see Supplementary Note 5 for the definition) of local polyhedra in bulk cubic diamond (cd) crystal over the pressure range from 0 to 15 GPa as well as local polyhedra centred on atoms with a coordination number (CN) of 4 in the low-density amorphous (LDA) form at 0 and 10 GPa relative to a perfect tetrahedron. **b** The distribution of RMSD of local polyhedra in bulk β-Sn crystal over the pressure range from 0 to 15 GPa as well as local polyhedra centred on atoms with CN = 6 in the high-density amorphous (HDA) form at 12 GPa and the very-high-density amorphous (VHDA) form at 15 GPa relative to a perfect local β-Sn polyhedron or a perfect octahedron. **c** The distribution of RMSD of local polyhedra in bulk simple hexagonal (sh) crystal over the pressure range from 10 to 15 GPa as well as local polyhedra centred on atoms with CN = 8 in the HDA form at 12 GPa and the VHDA form at 15 GPa relative to a perfect local sh polyhedron. When computing these distributions relative to four- or six-folded polyhedrons, 1,001 snapshots were used for the three amorphous forms after relaxing for 1 ps at the corresponding conditions, while only 101 and 26 snapshots were used for the HDA and VHDA forms, respectively, for the distributions relative to the eight-folded polyhedron because of its slower efficiency. The temperature considered here is always 300 K.

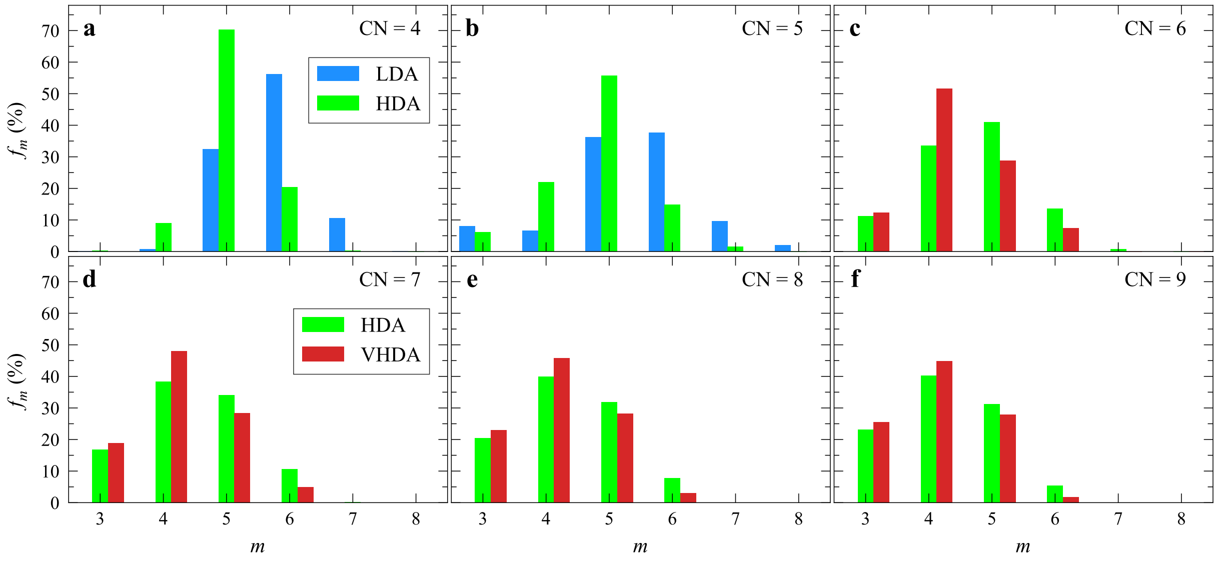

Supplementary Fig. 10. Ring analysis results for the three amorphous forms. The distribution of m-membered rings surrounding atoms with different coordination numbers (CN) for each of the three amorphous forms of silicon, namely, low-density amorphous (LDA), high-density amorphous (HDA) and very-high-density amorphous (VHDA). **a**-**f** correspond to atoms with a CN of from 4 to 9. Here, a cutoff of 2.85 Å was used to determine CN and whether there is a bond between a pair of atoms.

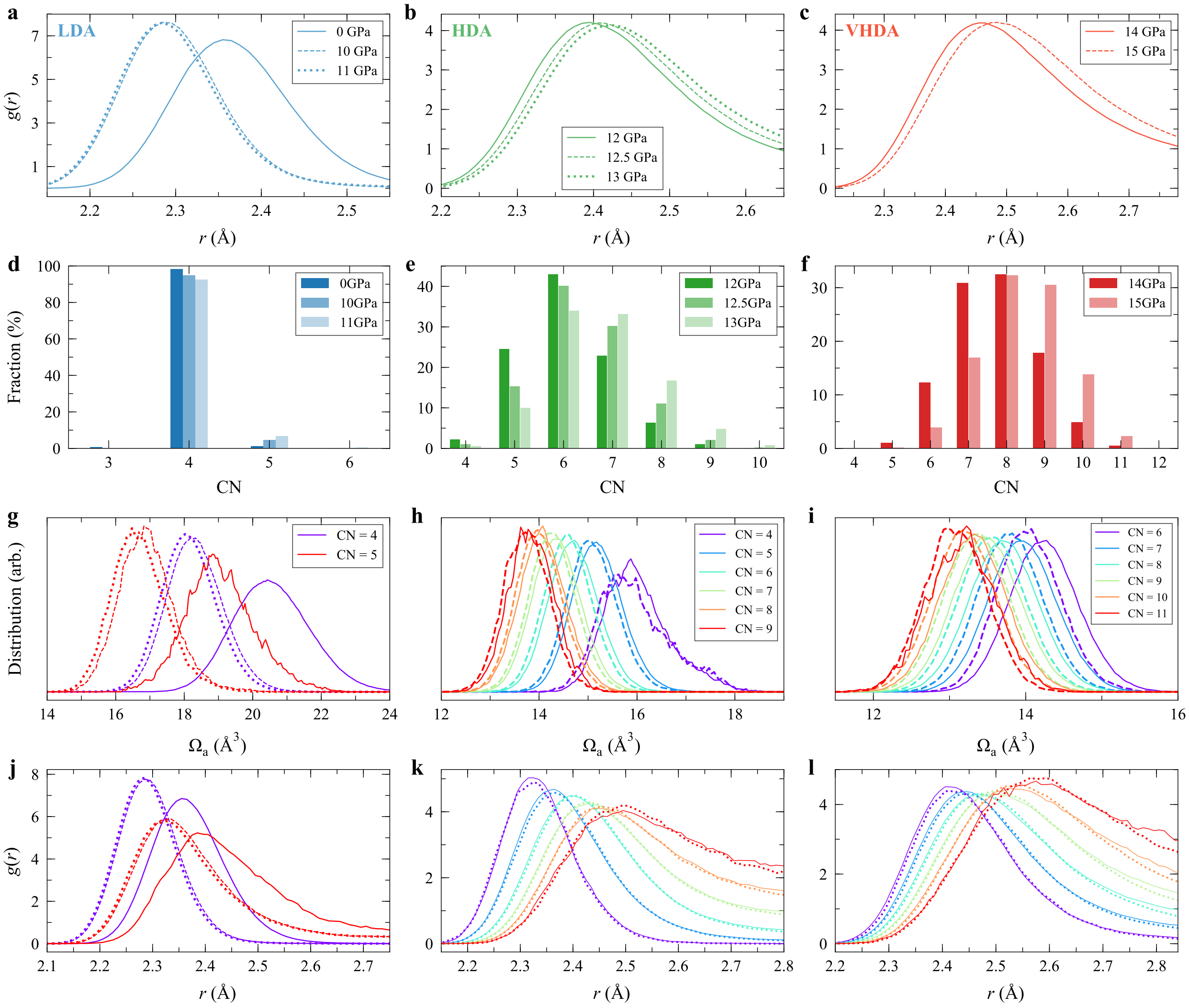

Supplementary Fig. 11. **The shift of the first peak of the radial distribution function with increasing hydrostatic pressure on the three amorphous forms.** **a**, **b**, and **c** show $g(r)$ of the low-density amorphous (LDA), high-density amorphous (HDA), and very-high-density amorphous (VHDA) forms, respectively, at different pressures (see legend). The temperature is always 300 K. The LDA sample at 0 GPa is the as-quenched sample. The LDA sample at 10 GPa was relaxed for 3 ns after increasing pressure linearly from 0 to 10 GPa within 100 ps. The LDA sample at 11 GPa was obtained after relaxing the $\mathrm{LDA}_{10,200}$ sample at 11 GPa for 2 ns. The HDA sample at 12 GPa is the $\mathrm{HDA}_{12,1000}$ sample. The HDA samples at 12.5 and 13 GPa have been relaxed at the corresponding pressure for 200 and 50 ps, respectively. The VHDA samples at 14 and 15 Pa have been relaxed at the corresponding pressure for 20 and 55 ps (case 1), respectively. **d**, **e**, and **f** are the distribution of coordination number (CN) for the LDA, HDA, and VHDA forms, respectively, at different pressures. **g**, **h**, and **i** are the distribution of atomic volume (Ω_a) for atoms with different CN in the LDA, HDA, and VHDA forms, respectively, at different pressures. **j**, **k**, and **l** are the radial distribution function $g(r)$ for atoms with different CN in the LDA, HDA, and VHDA forms, respectively, at different pressures. The solid, dashed, and dotted lines in **g-l** correspond to low, medium, and high pressures, respectively.

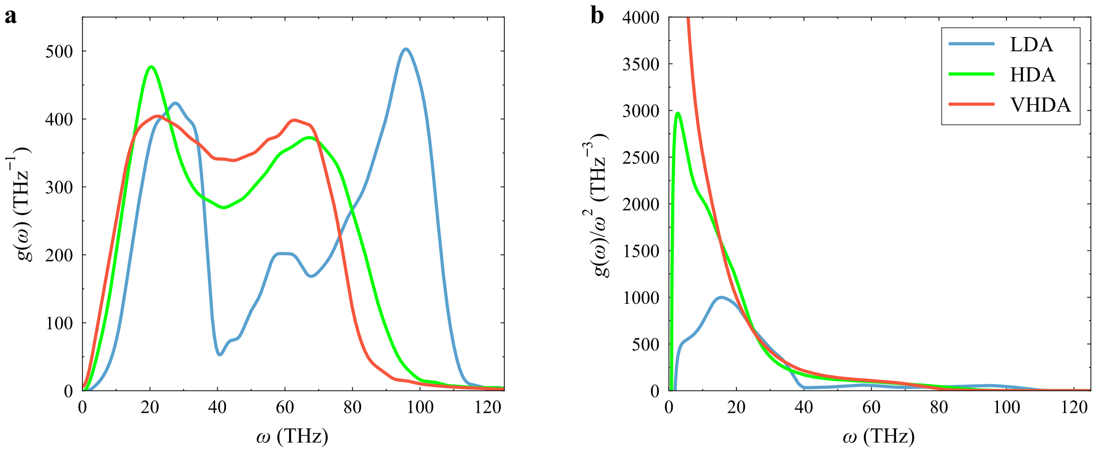

Supplementary Fig. 12. The vibrational density of states (VDOS) and reduced VDOS for the three different amorphous forms. **a** VDOS. **b** Reduced VDOS. These VDOSs were obtained through Fourier transformations of the velocity auto-correlation functions (VACFs). To ensure higher accuracy, we averaged 21 VACF curves over a given time window for each of the three glass forms. Specifically, for low-density amorphous (LDA), we evenly chose 21 states over the time interval of 200-400 ps during relaxation at 10 GPa after a linear pressure increase; for high-density amorphous (HDA), we used 21 states over the time interval of 1000-1200 ps during relaxation at 12 GPa after a linear pressure increase. The corresponding volume evolutions for the two time windows are shown in Fig. 1a. For very-high-density amorphous (VHDA), we used 21 states over the time interval of 30-50 ps during relaxing $LDA_{10,200}$ at 15 GPa (case 1) after a quick pressure increase. The corresponding volume evolution for this time window is shown in Supplementary Fig. 1a.

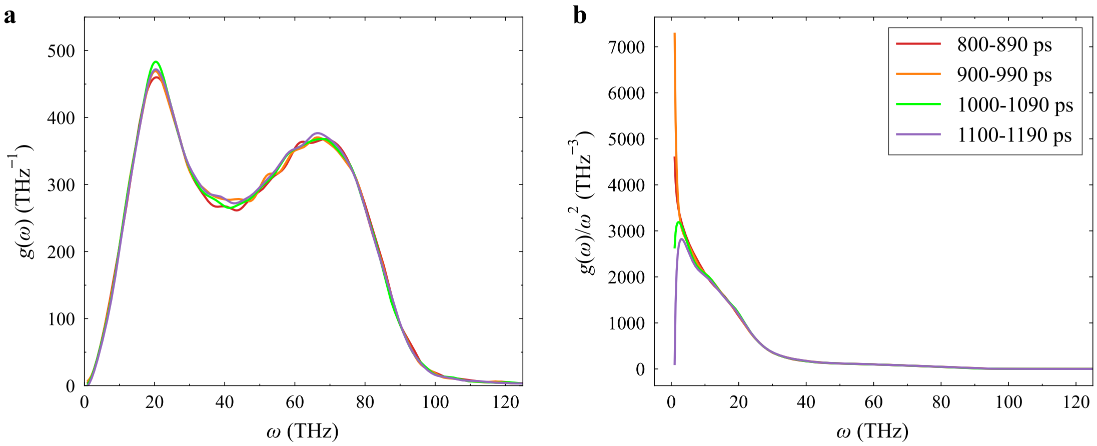

Supplementary Fig. 13. The temporal evolution of the vibrational density of states (VDOS) and reduced VDOS for high-density amorphous (HDA) during relaxation at 12 GPa after a linear pressure increase. a, b The time evolutions of VDOS and reduced VDOS, respectively. For each time window shown in the legend, 10 velocity auto-correlation functions (VACFs) were averaged before conducting Fourier transformations. In **b**, we can see that the quasi-elastic component decreases with time, indicating the stabilisation of HDA during the relaxation process.

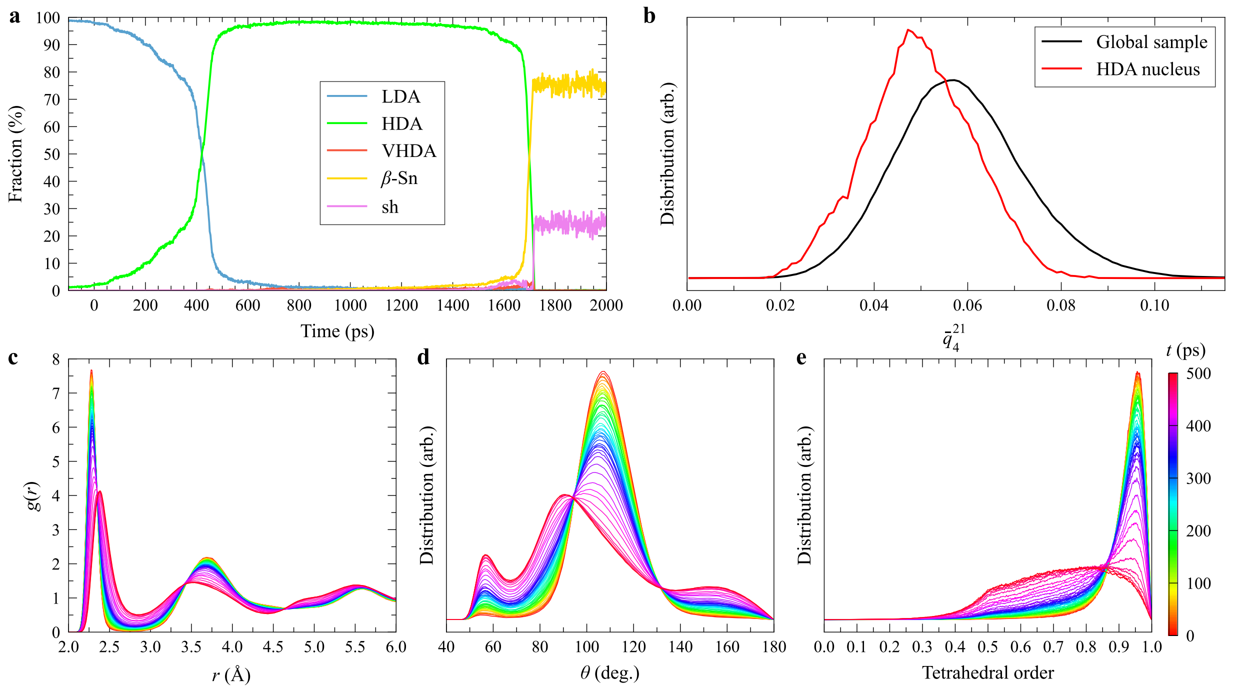

Supplementary Fig. 14. Further information about the low-density amorphous (LDA) to high-density amorphous (HDA) transition at 12 GPa. a The variation of the fraction of the five different local structural environments (LDA, HDA, very-high-density amorphous (VHDA), β-Sn and simple hexagonal (sh)) with time during the pressure jump from 0 to 12 GPa (over the time window between -100 and 0 ps) and the subsequent isothermal-isobaric relaxation at 12 GPa on the as-quenched sample. **b** shows the correlation between the location of HDA nucleation and the initial structure of the as-quenched sample. Specifically, the black and red curves represent the distributions of coarse-grained local bond orientational order parameter $\bar{q}_4^{21}$ of all atoms and the 151 atoms, which would constitute the largest HDA nucleus upon relaxation for 100 ps at 12 GPa, respectively, in the initial as-quenched sample at 0 GPa. 1,001 snapshots over 1 ps were used for these distributions. **c**, **d**, and **e** show the evolution of the radial distribution function $g(r)$, bond angular distribution function (BADF), and distribution of the tetrahedral order parameter, respectively, during the LDA-HDA transition at 12 GPa.

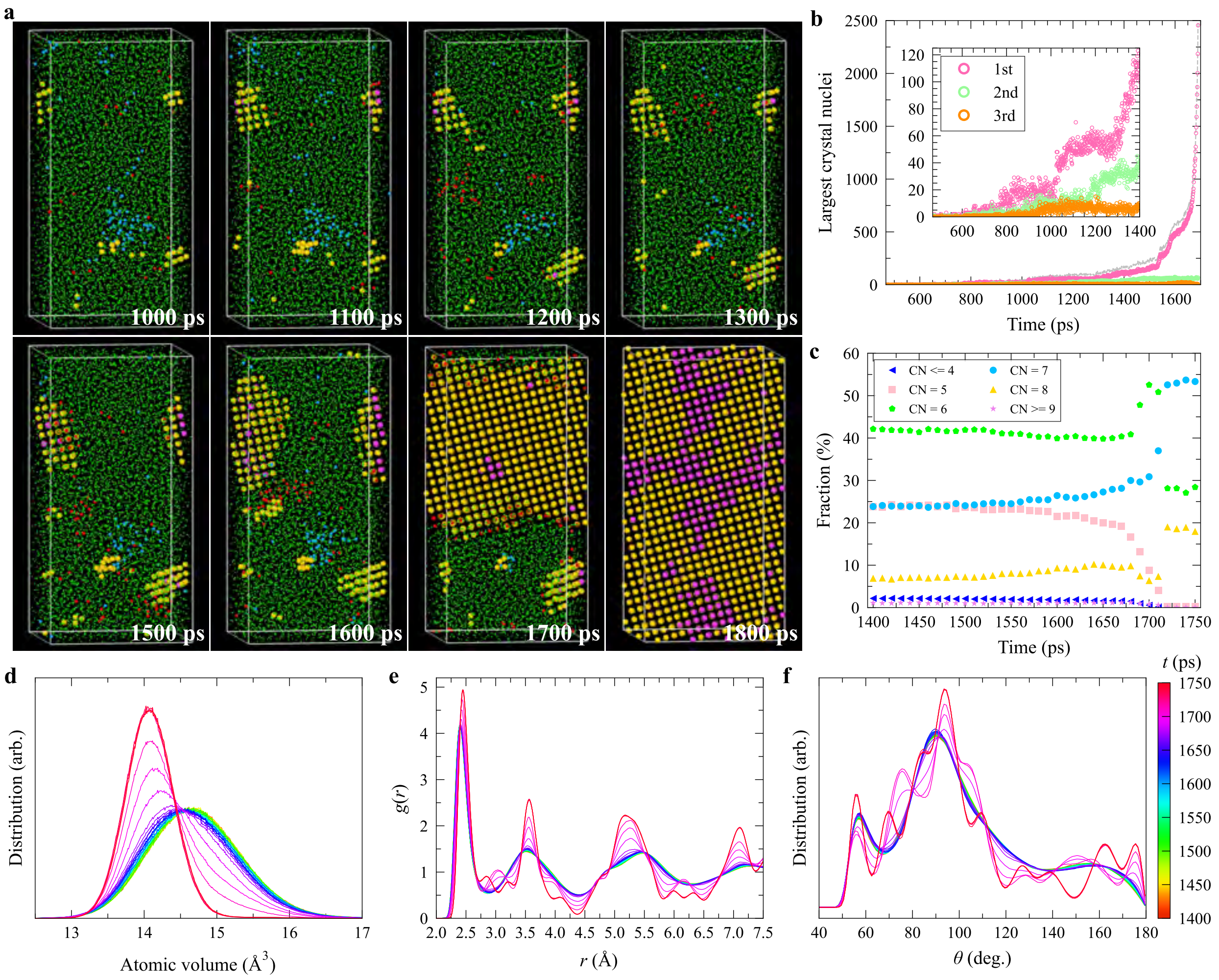

Supplementary Fig. 15. **Crystallisation behaviour of high-density amorphous (HDA).** This crystallisation occurs after the low-density amorphous (LDA) to HDA transition when relaxing the as-quenched a-Si sample at 12 GPa. **a** Typical structural snapshots during the crystallisation process. Blue, green, red, yellow, and magenta spheres represent LDA-, HDA-, very-high-density amorphous (VHDA)-, β-Sn-, and simple hexagonal (sh)-like atoms, respectively. The atom size is adjusted for different structural types for clarity. **b** The size evolution of the first three largest crystal nuclei (both β-Sn- and sh-like atoms are included) during the crystallisation process. The dashed silver line represents the total number of crystal-like atoms. The inset highlights the initial stage of the crystallisation process. A cutoff of 2.85 Å was used when conducting cluster analysis. **c**, **d**, **e**, and **f** show the variation of the coordination number (CN) distribution, atomic volume distribution, radial distribution function $g(r)$, and bond angle distribution function (BADF), respectively, during this transition.

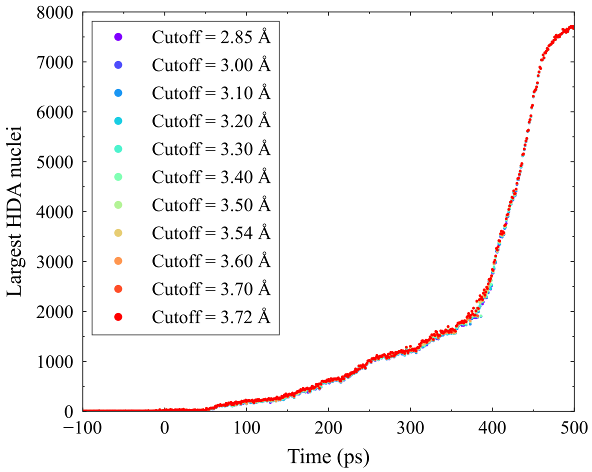

Supplementary Fig. 16. The independence of the cluster analysis outcomes from the chosen cutoff value. The size evolution of the first largest high-density amorphous (HDA) nuclei during the low-density amorphous (LDA) to HDA transition when relaxing the as-quenched a-Si sample at 12 GPa. Cluster analysis was employed to ascertain the cluster size, wherein a range of cutoff values spanning from 2.85 to 3.72 Å (corresponding to the second peak in the $g(r)$ distribution of the LDA sample) were tested.

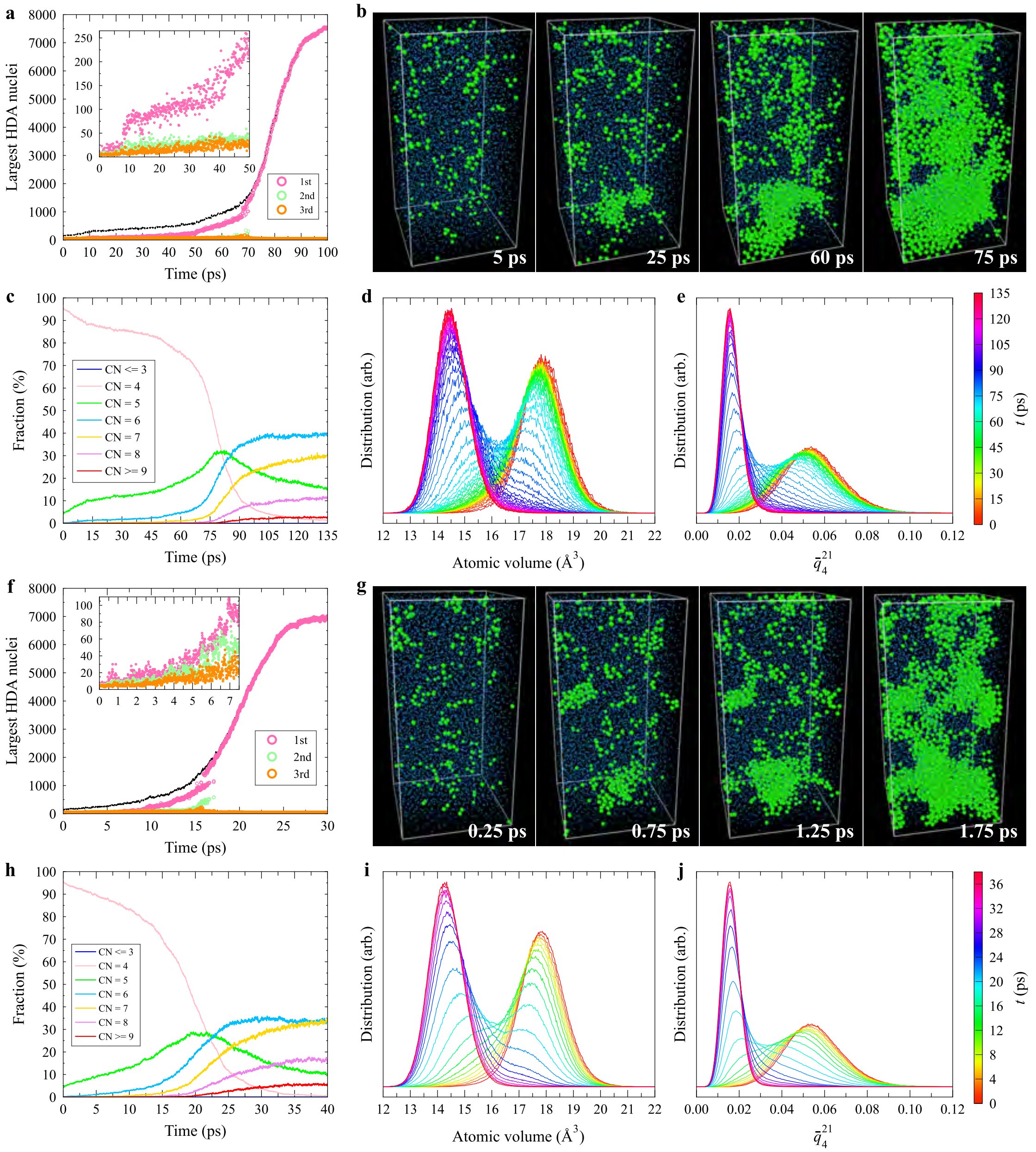

Supplementary Fig. 17. The low-density amorphous (LDA) to high-density amorphous (HDA) transition behaviours at 12.5 and 13 GPa. a-e and **f-j** are, respectively, for the LDA-HDA transitions occurring when relaxing the as-quenched a-Si sample at 12.5 and 13 GPa. The pressure, P, applied to the sample was increased quickly from 0 GPa to various target values at a constant rate of 10 GPa ps^{-1} before the isothermal-isobaric relaxation. **a** and **f** The size evolution of the first several largest HDA nuclei. The dashed line represents the total number of HDA-like atoms. The inset highlights the initial stage of the transition. A 2.85 Å cutoff was used for cluster analysis. **b** and **g** Typical structural snapshots during the LDA-HDA transitions. Blue and green spheres represent LDA- and HDA-like atoms, respectively. There is no other type of local atomic environment in the configurations exhibited here. The atom size is adjusted for different structural environments for clarity. **c**, **d**, and **e** (**h**, **i**, and **j**) show the variation of the coordination number (CN) distribution, atomic volume distribution, and distribution of coarse-grained local bond orientational order parameter $\bar{q}_4^{21}$, respectively, during the transitions.

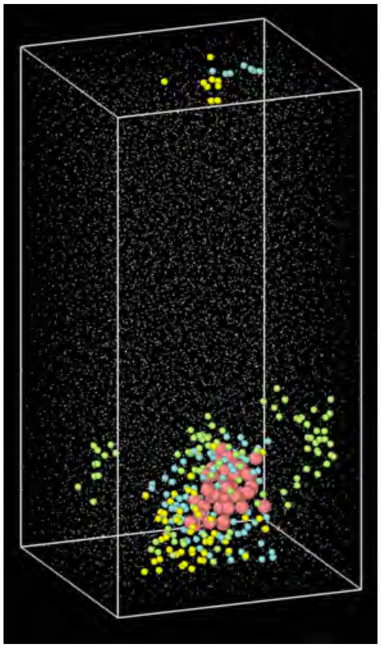

Supplementary Fig. 18. The nucleation sites during amorphous-amorphous transition (AAT) in silicon at room temperature are dictated by the initial structure. The snapshot of the initial a-Si sample at 0 GPa and 300 K. The atoms that later form the nuclei of high-density amorphous (HDA) structures are depicted across three distinct simulations. These simulations use the same initial state but employ different pressure increase protocols, including a linear pressure ramp from 0 to 12 GPa over 100 ps, as well as two instantaneous pressure jumps from 0 to 12 GPa, each initiated with a different set of initial velocity distributions. Within the context of the three simulations, the pink spheres represent the 42 atoms consistently present within the 151-atom HDA nuclei. These nuclei are shared among all three simulations, despite their different pressure increase protocols. Cyan, lime, and yellow spheres denote the remaining 109 atoms forming the 151-atom HDA nuclei in the three simulations, respectively. Small white spheres indicate atoms that do not participate in the composition of the 151-atom nuclei in any of the three cases. The atom sizes have been adjusted to enhance clarity.

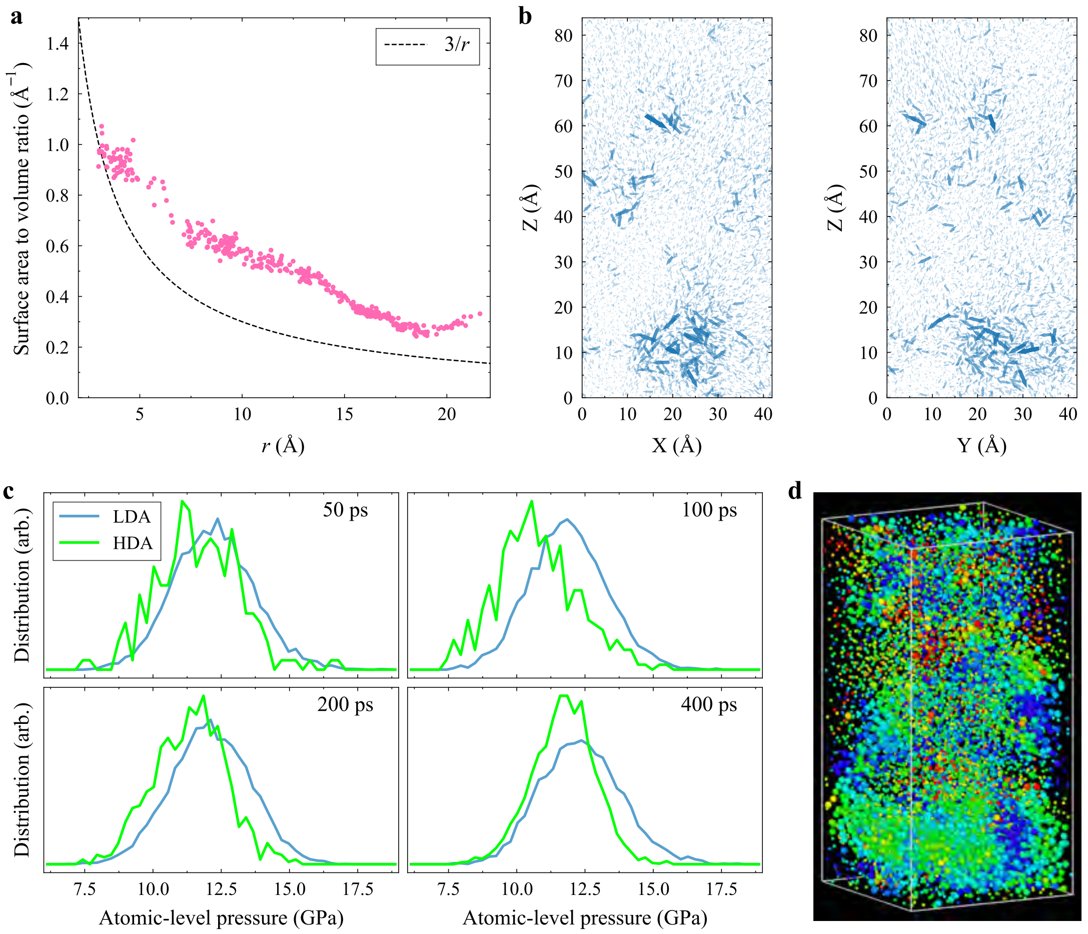

Supplementary Fig. 19. Mechanical features of amorphous-amorphous transition (AAT). a The relationship between the surface area-to-volume ratio of the largest high-density amorphous (HDA) nucleus and its radius (derived from the volume, assuming nuclei are spherical) during the initial 400 ps of relaxation of the as-quenched a-Si sample at 12 GPa, subsequent to a linear pressure ramp within 100 ps. The black dashed curve represents the surface area-to-volume ratio of an ideal sphere ($3/r$) as a function of r. The surface area of the HDA nuclei was computed using the Gaussian blurring of the density field, while the volume was determined by summing the atomic volumes of the constituent atoms within the HDA nuclei. This atomic volume calculation was made through Voronoi analysis. These calculations were performed using the OVITO software [11]. **b** The projection on the XZ (left) and YZ (right) planes of the displacement field during the HDA nucleation-growth process (the atomic positions of the snapshot at 100 ps minus those of the snapshots at 0 ps during the isothermal-isobaric relaxation at 12 GPa). Both the width and transparency of the displacement vectors are scaled with their length. The largest displacement over the 100 ps is 3.94 Å. **c** The distribution of atomic-level pressure after conducting coarse-grained up to neighbour atoms three times, at four time points (denoted on each panel) during the isothermal-isobaric relaxation at 12 GPa after a linear pressure ramp within 100 ps. **d** The spatial distribution of the atomic-level pressure (coarse-grained up to neighbour atoms three times to reduce the noise) at 400 ps corresponding to the one in Fig. 2b. The small and large spheres correspond to low-density amorphous (LDA)- and HDA-like atoms, respectively.

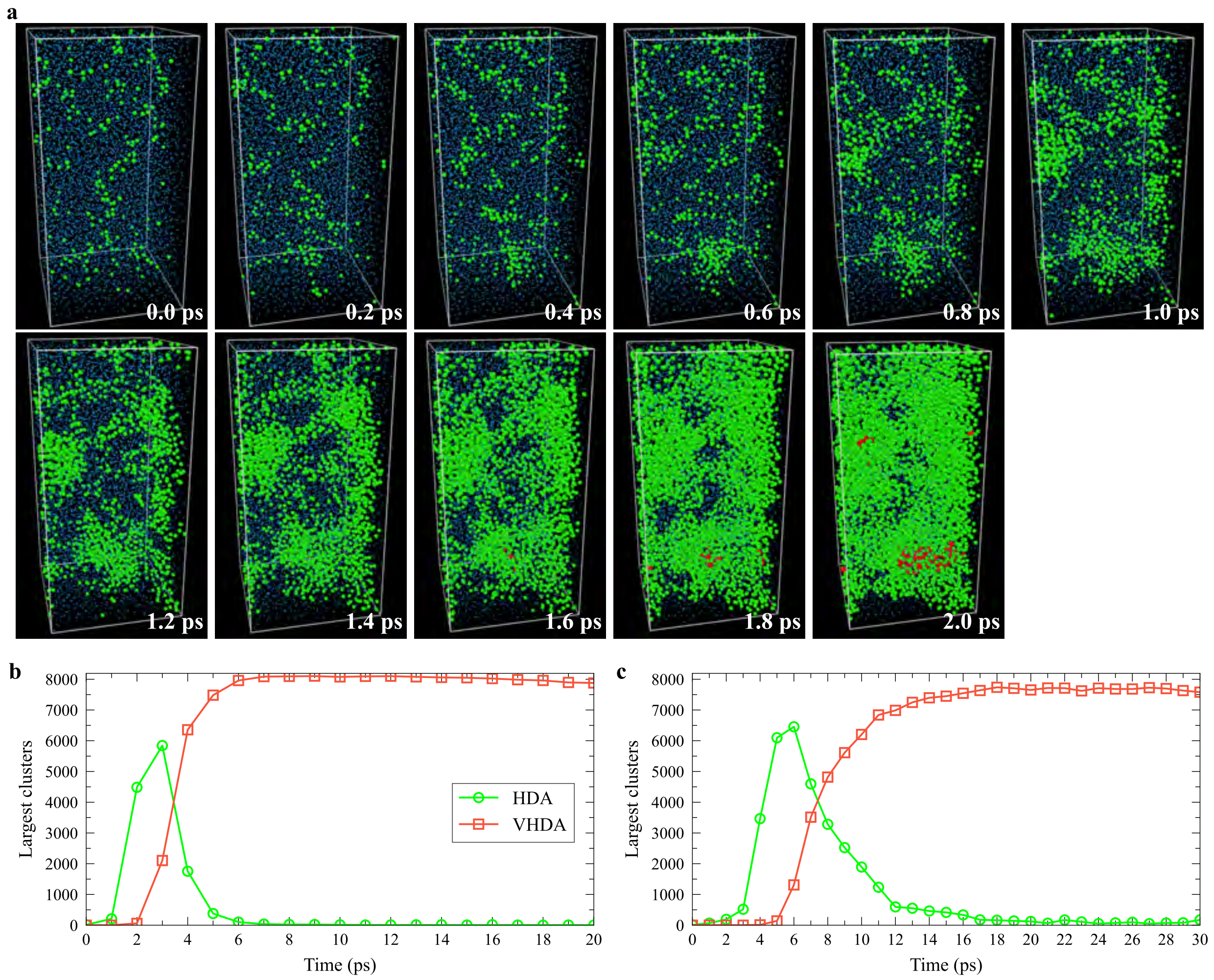

Supplementary Fig. 20. More information on low-density amorphous (LDA) to very-high-density amorphous (VHDA) transitions. **a** The structural snapshots regarding the formation of high-density amorphous (HDA) clusters in the LDA-VHDA transition when relaxing the $LDA_{10,200}$ sample at 15 GPa (case 1). Blue, green, and red spheres represent LDA-, HDA-, and VHDA-like atoms. The atom size is adjusted for different structural types for clarity. There is no crystal-like atom over the time window considered here. **b** and **c** show the size evolution of the largest HDA and VHDA clusters during the LDA-VHDA transition when relaxing the $LDA_{10,200}$ sample at 15 GPa (case 0) and 14 GPa, respectively.

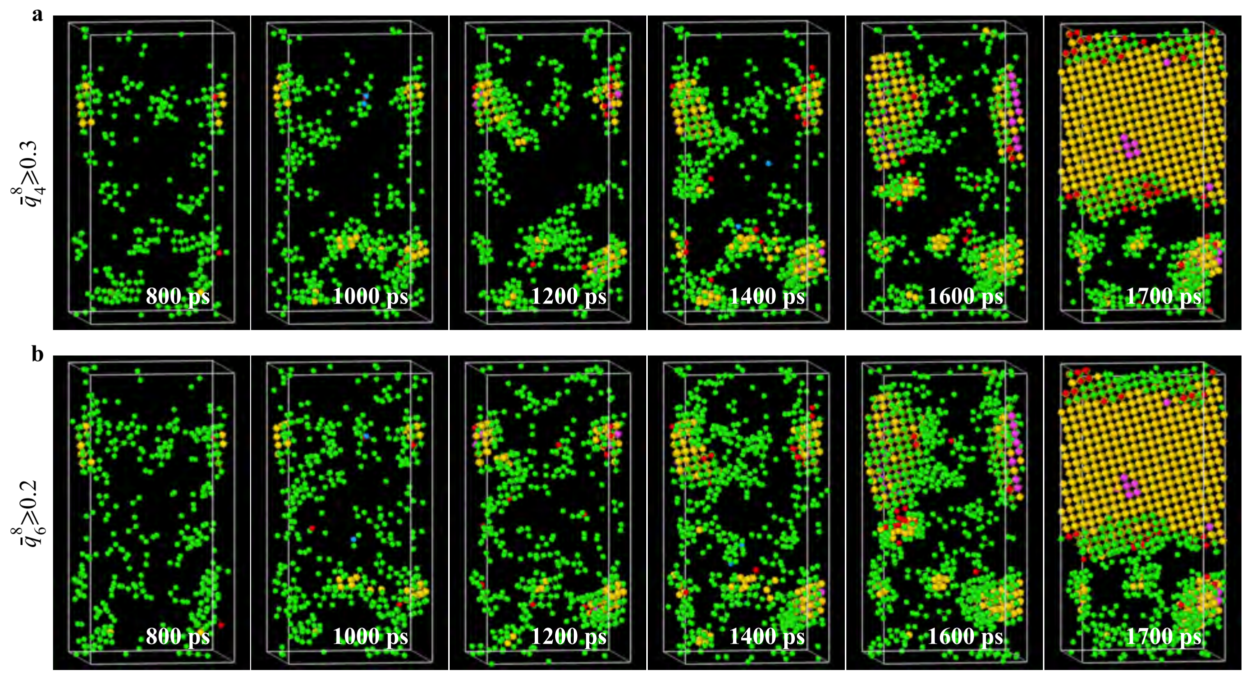

Supplementary Fig. 21. Precursor of β-Sn crystal. The structural snapshots during the crystallisation process after the low-density amorphous (LDA) to high-density amorphous (HDA) transition when relaxing the as-quenched a-Si sample at 12 GPa. Blue, green, red, yellow, and magenta spheres represent LDA-, HDA-, very-high-density amorphous (VHDA)-, β-Sn-, and simple hexagonal (sh)-like atoms, respectively. The atom size is adjusted for different structural types for clarity. Only atoms with $\bar{q}_4^8 \geqslant 0.3$ are shown in panel **a** while only atoms with $\bar{q}_6^8 \geqslant 0.2$ are shown in panel **b**.

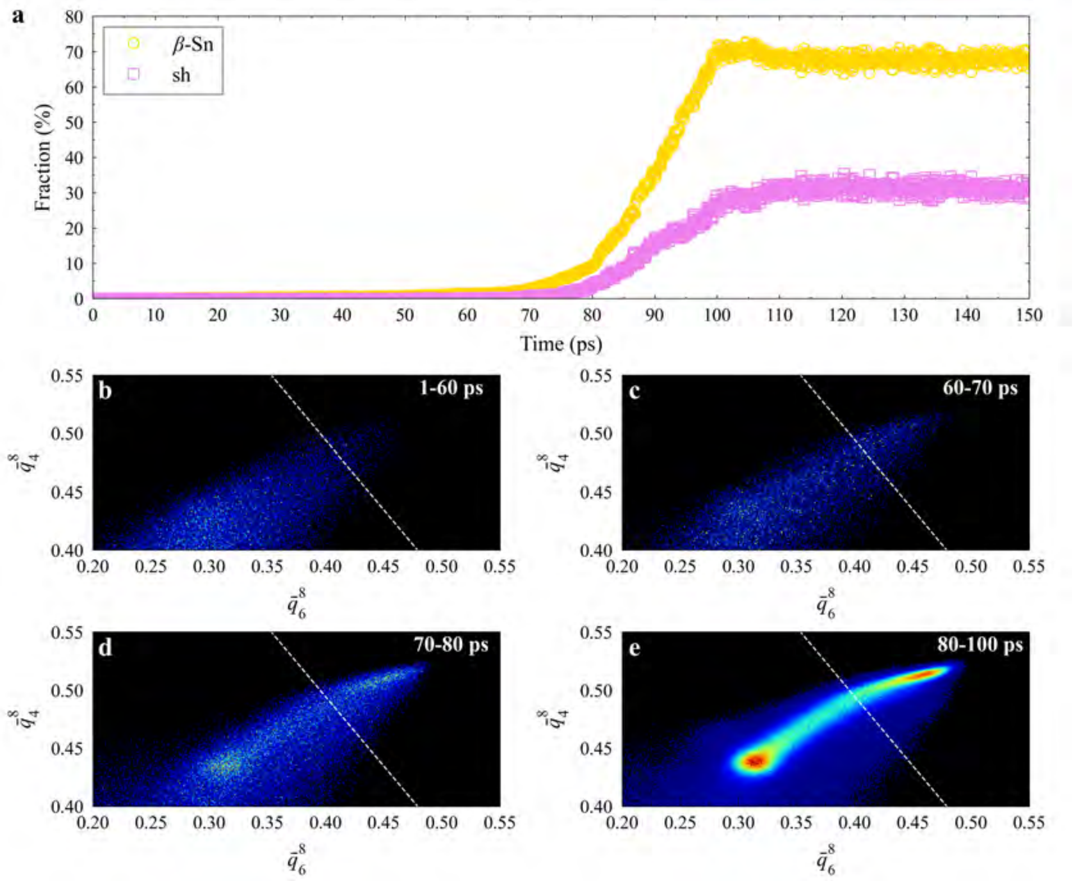

Supplementary Fig. 22. **A two-step crystallisation process.** **a** shows the temporal change of the fraction of β-Sn- and simple hexagonal (sh)-like local environments when annealing low-density amorphous (LDA) sample at 15 GPa (case 1). **b-e** show the order-parameter distributions of local crystalline environments on the $\bar{q}_4^8$-$\bar{q}_6^8$ plane at four time-windows (denoted on each panel) for the crystallisation process shown in **a**. Red (blue) colour corresponds to high (low) density.

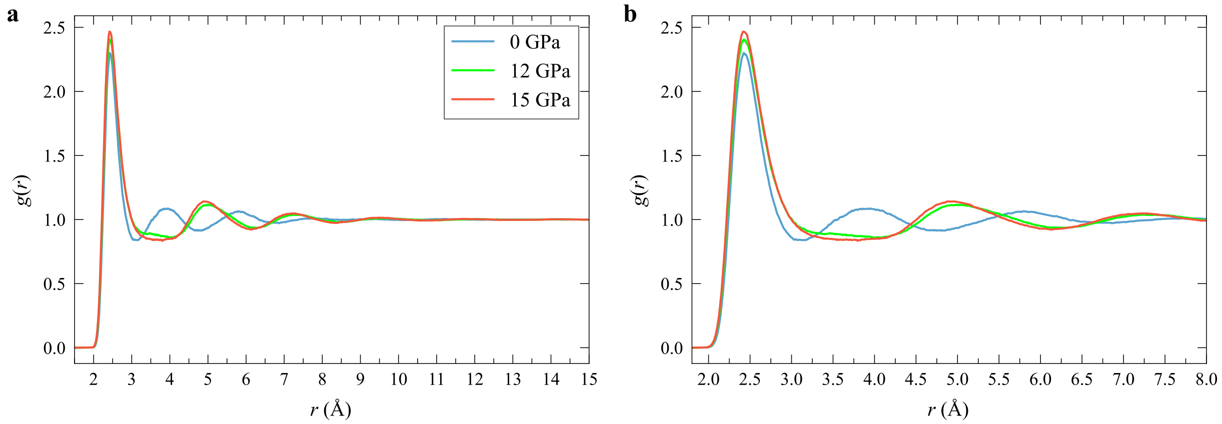

Supplementary Fig. 23. Structural features of liquid Si at different pressures. a Radial distribution function $g(r)$ of liquid silicon at 1500 K but at different pressures. The sample at 0 GPa was obtained through melting cubic-diamond crystal at high temperature; The samples at 12 and 15 GPa were obtained through heating high-density amorphous ($\mathrm{HDA}_{12,1000}$) and very-high-density amorphous ($\mathrm{VHDA}_{15,10}$) samples, respectively, at the corresponding pressure. **b** A zoom-up of **a** to highlight the short-range order.

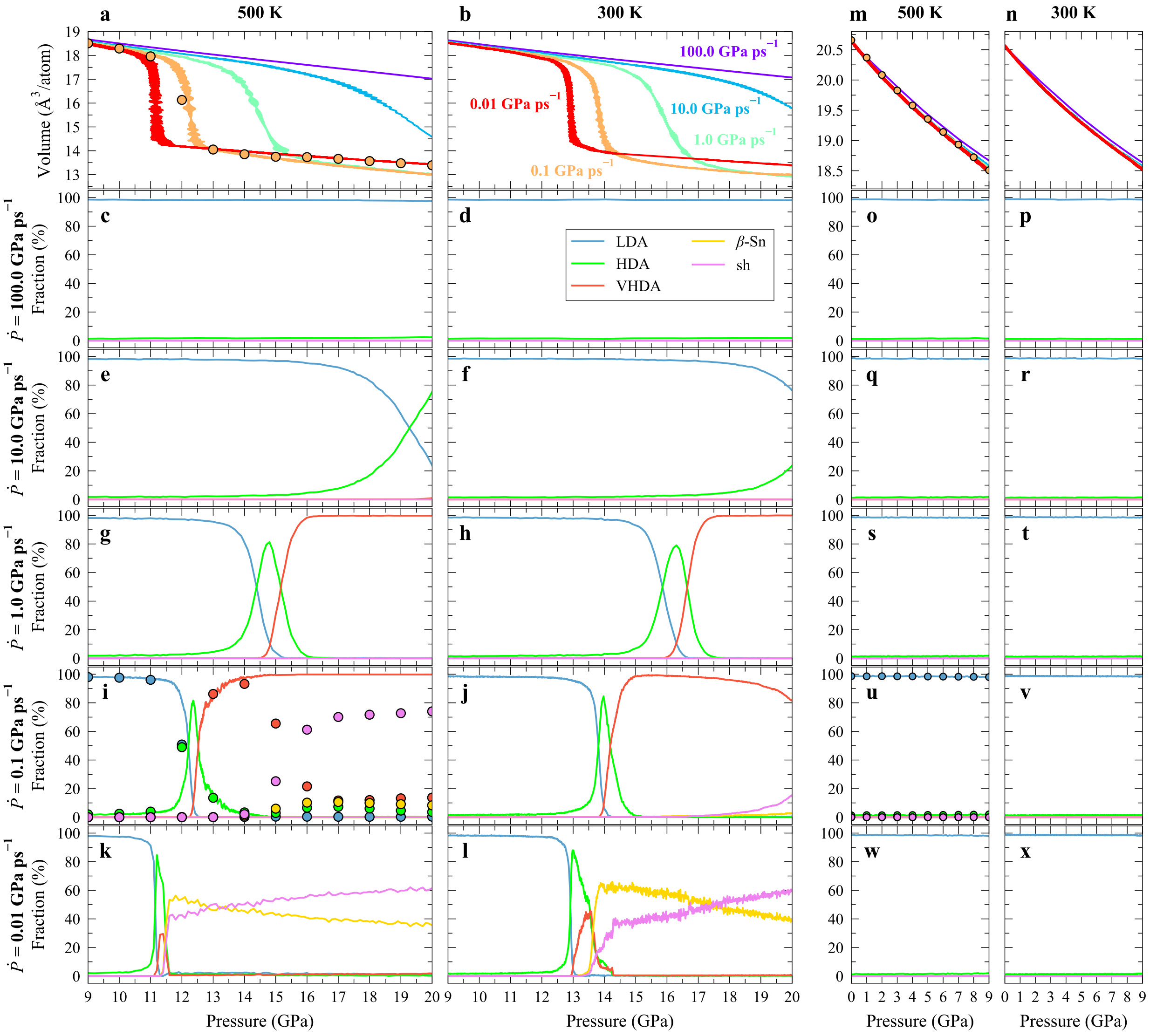

Supplementary Fig. 24. Pressurisation rate and temperature dependence of structural transition in a-Si. **a** and **b** show the volume variation of a-Si in the pressure range between 9 and 20 GPa when increasing hydrostatic pressure P from 0 to 20 GPa with five different rates at the temperature of 500 and 300 K, respectively. Each panel among **c**-**l** shows the variation of the fraction of the five different local structural environments (low-density amorphous (LDA), high-density amorphous (HDA), very-high-density amorphous (VHDA), β-Sn, and simple hexagonal (sh)) for each given loading condition. The figures from the upper to lower of the last five rows correspond to the rates of 100.0, 10.0, 1.0, 0.1, and 0.01 GPa ps^{-1}, respectively. The first (second) column corresponds to 500 (300) K. All lines in the plots represent the behaviours of the a-Si model generated in the current work, and the solid circles represent the evolution of the ultra-big a-Si sample generated in ref. [1]. The corresponding variations in the pressure range from 0 to 9 GPa are displayed in **m**-**x**.

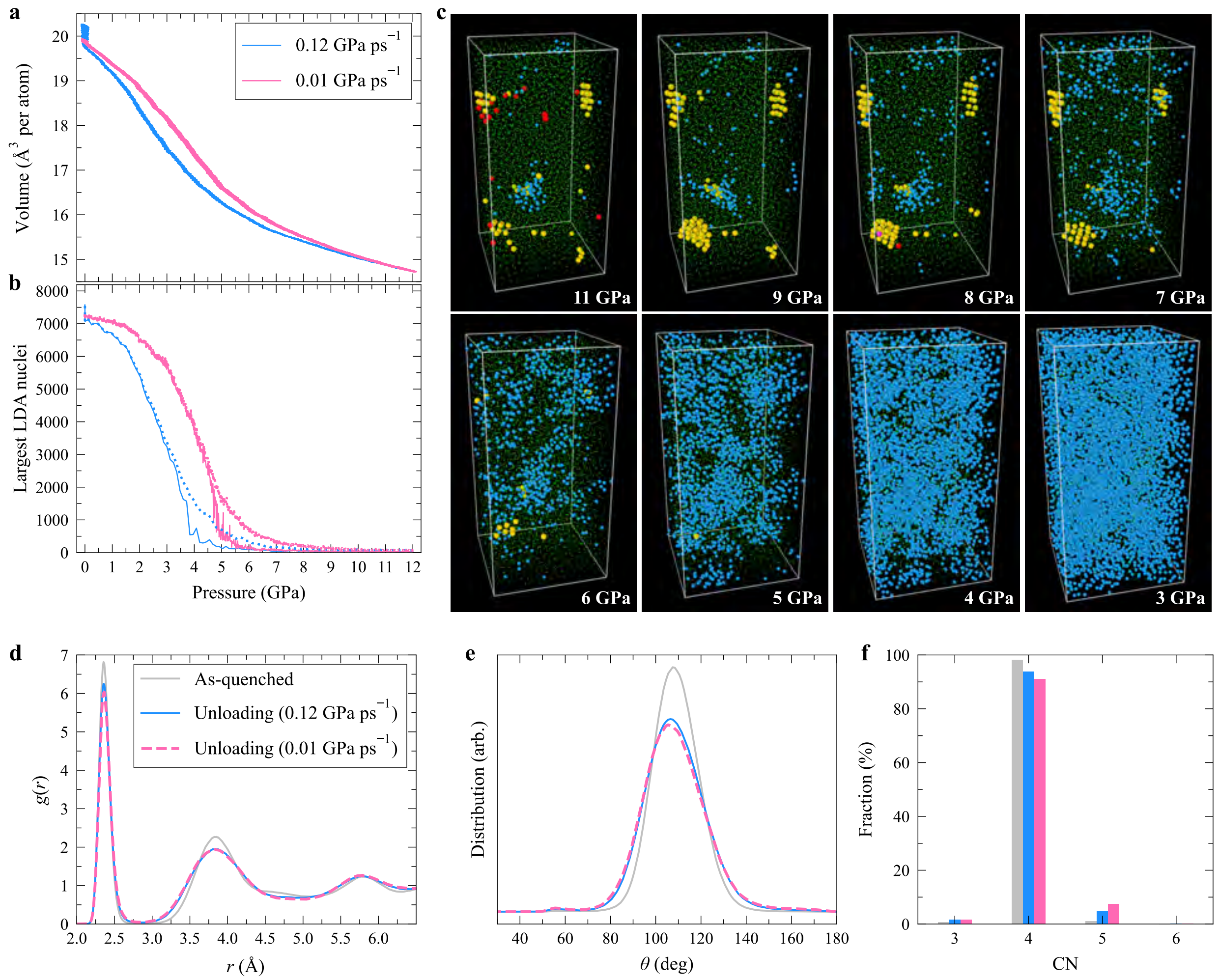

Supplementary Fig. 25. Structural change upon releasing pressure continuously on the high-density amorphous (HDA) form. **a** The variation of the average atomic volume when releasing pressure on the $HDA_{12,1000}$ sample using two different rates (see legend) at 300 K. The sample was relaxed for 1.2 ns (0.1 ns) after the pressure decreased to 0 GPa at a rate of 0.12 GPa ps^{-1} (0.01 GPa ps^{-1}). **b** The corresponding variation of the largest low-density amorphous (LDA) cluster size (solid lines) and the total number of LDA-like atoms (dotted lines) with decreasing pressure. **c** The structural snapshots while releasing pressure at a rate of 0.01 GPa ps^{-1}. Blue, green, red, yellow, and magenta spheres represent LDA-, HDA-, very-high-density amorphous (VHDA)-, β-Sn-, and simple hexagonal (sh)-like atoms, respectively. The atom size is adjusted for different structural types for clarity. **d-f** The radial distribution function $g(r)$, bond angle distribution function (BADF), and distribution of coordination number (CN) of samples after unloading pressure on the $HDA_{12,1000}$ sample using the two different rates as well as the initial as-quenched a-Si sample at 0 GPa and 300 K.

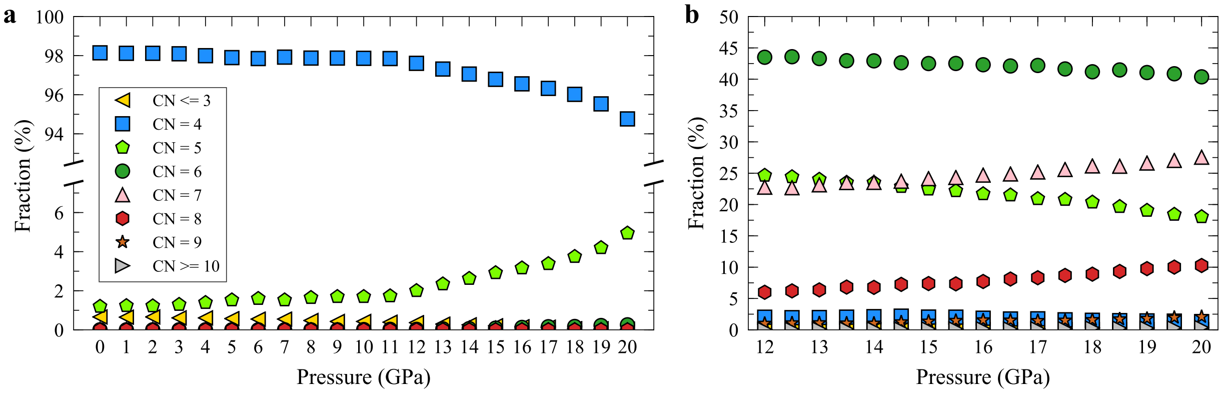

Supplementary Fig. 26. Pressure dependence of the coordination number (CN). a The variation of CN distribution of the low-density amorphous (LDA) sample when increasing pressure on the initial as-quenched sample at a constant rate of 100 GPa ps^{-1} and 300 K. From 0 to 20 GPa, the fraction of atoms with CN = 4 decreases from 98.1% to 94.8%. **b** The variation of the CN distribution of the high-density amorphous (HDA) sample when increasing pressure on the $HDA_{12,1000}$ sample at a constant rate of 100 GPa ps^{-1} and 300 K. From 12 to 20 GPa, the fraction of atoms with CN = 6 decreases from 43.5% to 40.4%. Here, a constant cutoff of 2.85 Å was used to determine CN.

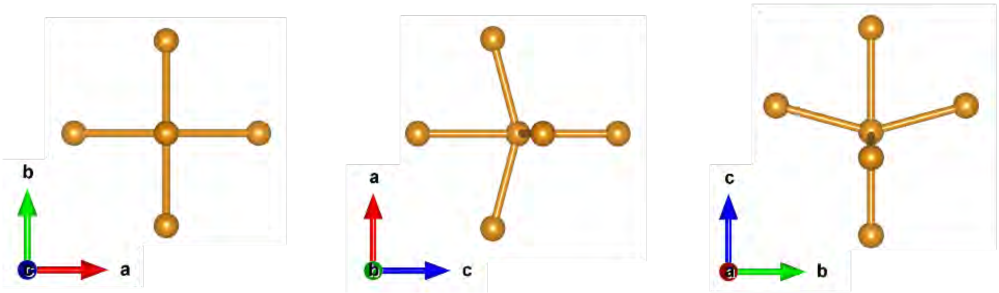

Supplementary Fig. 27. Three perspectives of the perfect local polyhedron in a β-Sn crystal.

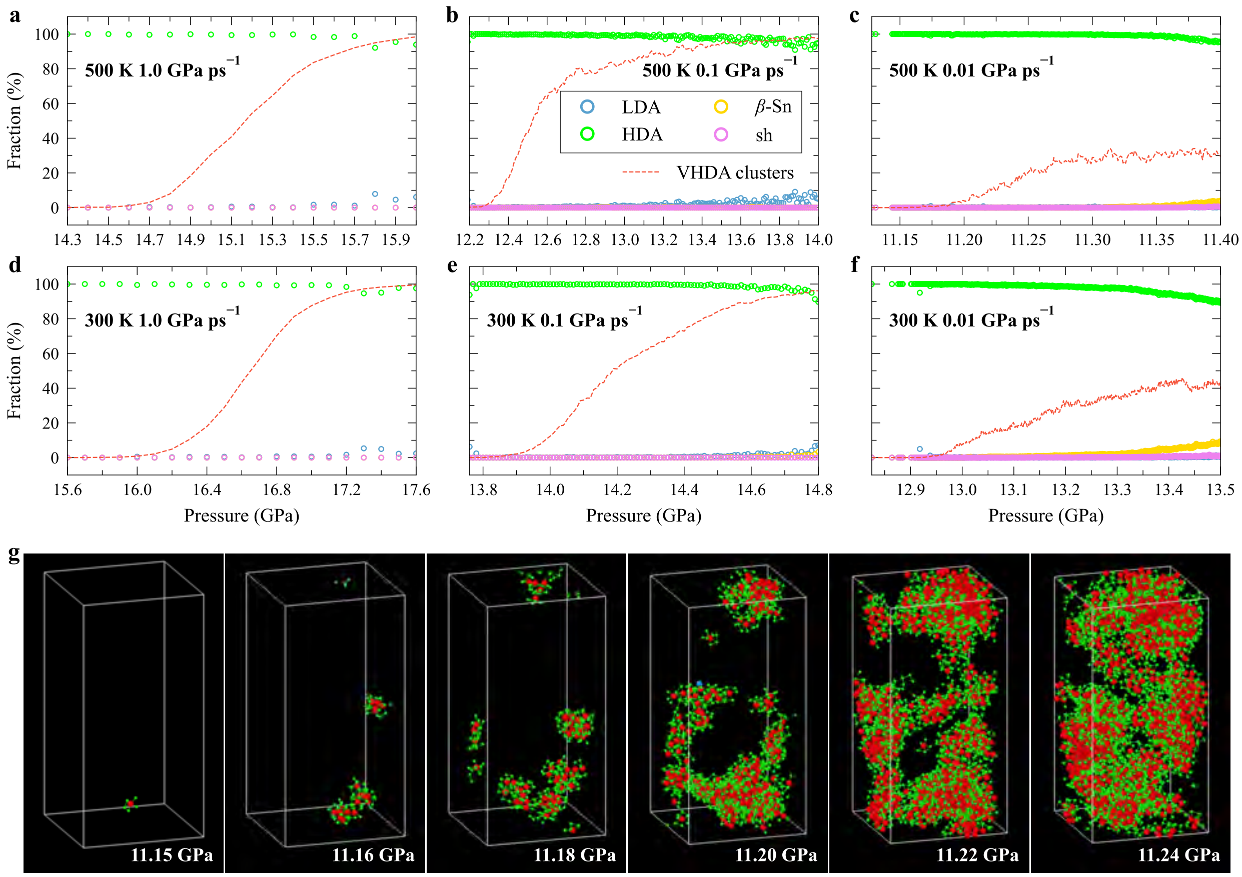

Supplementary Fig. 28. The low-density amorphous (LDA) to very-high-density amorphous (VHDA) transition behaviours during increasing pressure continuously. **a-f** The structural environment of the first neighbouring shell surrounding VHDA-like individual atoms and clusters during the emergence of VHDA-like atoms when compressing the as-quenched a-Si model from 0 to 20 GPa at different conditions (denoted on each panel). The circles with different colours (see legend) represent the fraction of atoms in different local structural environments within the first (both outer and interior) neighbouring shell surrounding VHDA-like atoms at a given pressure. The red dashed line represents the fraction of VHDA-like atoms relative to all atoms in the entire supercell. We confirmed that most crystal-like atoms belong to neighbouring interior shells through visualisation. **g** Visualisation of the structural environment of the first neighbouring shell surrounding VHDA-like individual atoms and clusters during the emergence of VHDA-like atoms when compressing the as-quenched a-Si model from 0 to 20 GPa at 500 K at a constant rate of 0.01 GPa ps^{-1}. Only VHDA-like atoms and their first neighbours are shown in each snapshot. Blue, green, red, yellow, and magenta spheres represent LDA-, high-density amorphous (HDA)-, VHDA-, β-Sn-, and simple hexagonal (sh)-like atoms, respectively. The atom size is adjusted for different structural types for clarity.

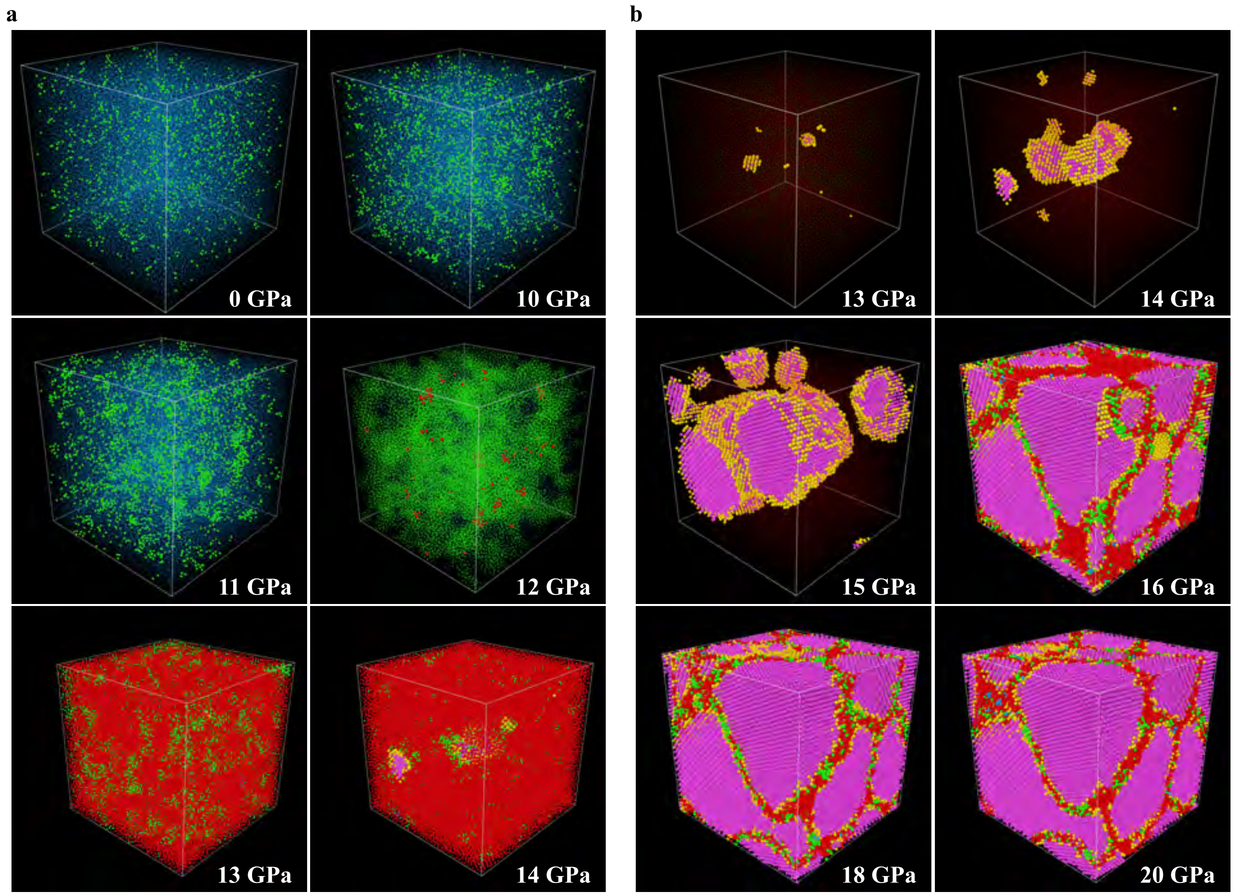

Supplementary Fig. 29. Amorphous-amorphous transition (AAT) and crystallisation in an ultra-big a-Si sample. Visualisation of the structural snapshots during the AAT (**a**) and crystallisation processes (**b**) when increasing hydrostatic pressure on an ultra-big a-Si sample (100,000 atoms) at 500 K from 0 to 20 GPa with a rate of 0.1 GPa ps^{-1}. These configurations in ref. [1] were analysed using the local structural order parameters developed in the current work. Blue, green, red, yellow, and magenta spheres represent low-density amorphous (LDA)-, high-density amorphous (HDA)-, very-high-density amorphous (VHDA)-, β-Sn-, and simple hexagonal (sh)-like atoms, respectively. The atom size is adjusted for different structural types for clarity.

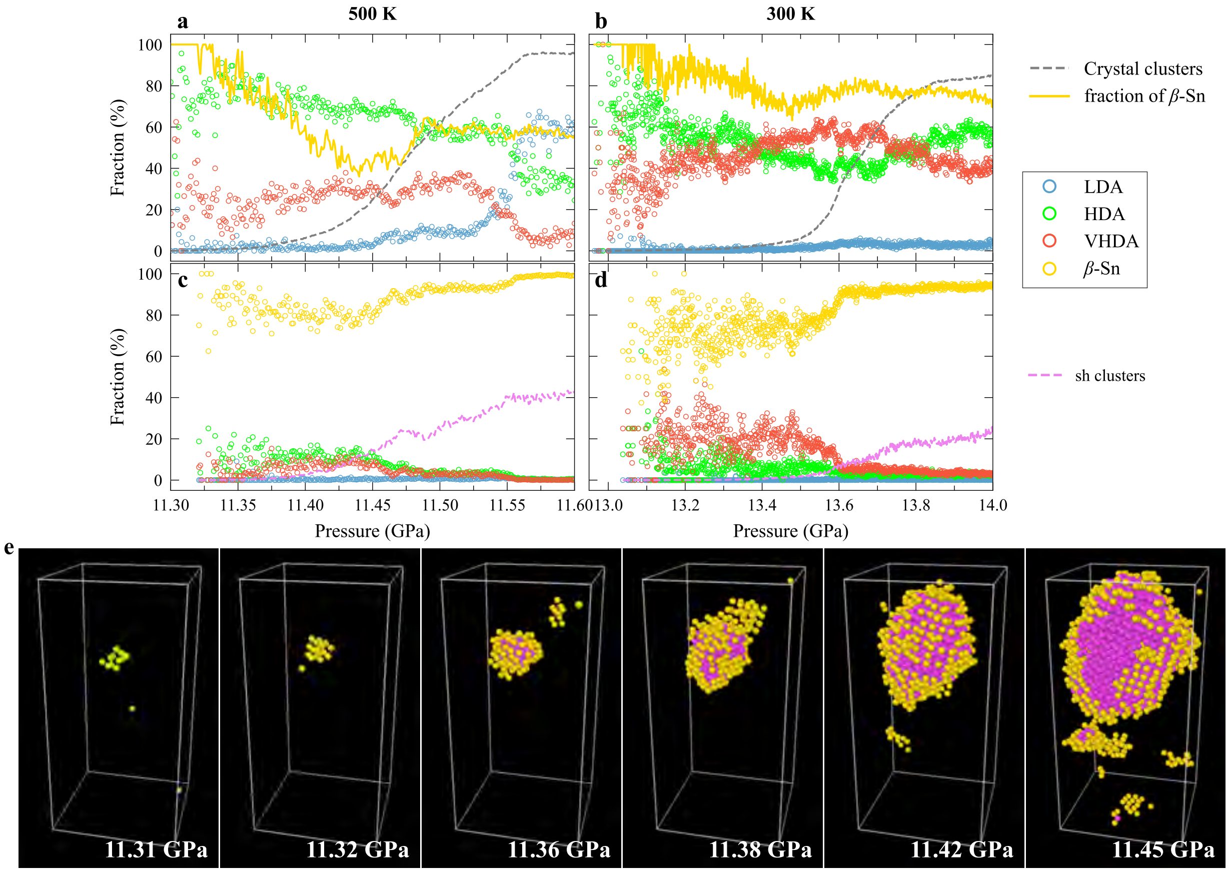

Supplementary Fig. 30. **Crystallisation processes induced by continuously increasing pressure on a-Si. a-d** The structural environment of the first neighbouring shell surrounding crystal-like individual atoms and clusters during crystallisation when compressing the as-quenched a-Si model from 0 to 20 GPa using a constant rate of 0.01 GPa ps^{-1} at both 500 K (left column) and 300 K (right column). The circles with different colours (see legend) represent the fraction of atoms in different local structural environments (low-density amorphous (LDA), high-density amorphous (HDA), very-high-density amorphous (VHDA) and β-Sn) within the first (both outer and interior) neighbouring shell surrounding crystal-like atoms at a given pressure. Both β-Sn- and simple hexagonal (sh)-like atoms are considered in the upper row, and only sh-like atoms are considered in the lower row. The silver dashed line in the upper row represents the fraction of both β-Sn- and sh-like atoms relative to all the supercell atoms, and the solid yellow line represents the fraction of β-Sn-like atoms among all crystal-like atoms. The magenta dashed line in the lower row represents the fraction of sh-like atoms relative to all the supercell atoms. **e** Visualisation of the structural snapshots during crystal nucleation and growth when increasing hydrostatic pressure on the as-quenched a-Si sample from 0 to 20 GPa using a rate of 0.01 GPa ps^{-1} at 500 K. Only crystal-like atoms are shown here. Yellow and magenta spheres represent β-Sn- and sh-like atoms, respectively.

III. Supplementary Tables 1-2

Supplementary Table 1. The best N that can minimize the overlap in the distributions of $\bar{q}_l^N$ between high-density amorphous (HDA) and very-high-density amorphous (VHDA) states, $O_{\mathrm{HDA-VHDA}}$, as well as the corresponding value of $O_{\mathrm{HDA-VHDA}}$ for each integer l over the range from 1 to 15. They are sorted based on $O_{\mathrm{HDA-VHDA}}$.

l	N	$O_{\mathrm{HDA-VHDA}}$
10	13	0.3634
12	11	0.4468
4	16	0.5271
6	28	0.6533
9	10	0.6544
11	16	0.7275
13	13	0.7281
8	8	0.7550
14	15	0.7641
5	20	0.8032
2	5	0.8121
7	16	0.8260
3	12	0.8467
1	5	0.8744
15	9	0.8964

Supplementary Table 2. The spherical coordinates of the first six nearest neighbours of a typical atom in a perfect bulk crystal of β-Sn structure at 300 K and 12 GPa.

r(Å)	$\theta(^\circ)$	$\phi(^\circ)$
2.43	180	105.3
2.43	-90	74.7
2.43	90	74.7
2.43	0	105.3
2.56	0	180
2.56	0	0

IV. Supplementary References

[1] Deringer, V. L. *et al.* Origins of structural and electronic transitions in disordered silicon. *Nature* **589**, 59–64 (2021).

[2] Deb, S. K., Wilding, M., Somayazulu, M. & McMillan, P. F. Pressure-induced amorphization and an amorphous–amorphous transition in densified porous silicon. *Nature* **414**, 528–530 (2001).

[3] McMillan, P. F., Wilson, M., Daisenberger, D. & Machon, D. A density-driven phase transition between semiconducting and metallic polyamorphs of silicon. *Nature Mater* **4**, 680–684 (2005).

[4] Morishita, T. High Density Amorphous Form and Polyamorphic Transformations of Silicon. *Phys. Rev. Lett.* **93**, 055503 (2004).

[5] Durandurdu, M. & Drabold, D. A. Ab initio simulation of first-order amorphous-to-amorphous phase transition of silicon. *Phys. Rev. B* **64**, 014101 (2001).

[6] LeCun, Y., Bengio, Y. & Hinton, G. Deep learning. *Nature* **521**, 436–444 (2015).

[7] Fan, Z. & Ma, E. Predicting orientation-dependent plastic susceptibility from static structure in amorphous solids via deep learning. *Nature Communications* **12**, 1506 (2021).

[8] Russo, J., Romano, F. & Tanaka, H. New metastable form of ice and its role in the homogeneous crystallization of water. *Nature Mater* **13**, 733–739 (2014).

[9] Demkowicz, M. J. & Argon, A. S. High-Density Liquidlike Component Facilitates Plastic Flow in a Model Amorphous Silicon System. *Phys. Rev. Lett.* **93**, 025505 (2004).

[10] Fan, Z., Ding, J., Li, Q.-J. & Ma, E. Correlating the properties of amorphous silicon with its flexibility volume. *Phys. Rev. B* **95**, 144211 (2017).

[11] Stukowski, A. Visualization and analysis of atomistic simulation data with OVITO–the Open Visualization Tool. *Modelling Simul. Mater. Sci. Eng.* **18**, 015012 (2010).
