# Supplementary material for: Microscopic mechanisms of pressure-induced amorphous-amorphous transitions and crystallisation in silicon
Source: Nat Commun. 2024 Jan 16;15:368. doi: 10.1038/s41467-023-44332-6 (PMC10792069; doi:10.1038/s41467-023-44332-6)
Supplement: Supplementary file 3 — Description of Additional Supplementary Files [file 41467_2023_44332_MOESM3_ESM.docx]

**Description of Additional Supplementary Files**

**Supplementary Data:** In this Supplementary Data, both the initial and final configurations of molecular dynamics simulations for isothermal-isobaric relaxations at 10, 12, and 15 GPa are provided.
